# RA-XII inhibits tumour growth and metastasis in breast tumour-bearing mice via reducing cell adhesion and invasion and promoting matrix degradation

**DOI:** 10.1038/srep16985

**Published:** 2015-11-23

**Authors:** Hoi-Wing Leung, Si-Meng Zhao, Grace Gar-Lee Yue, Julia Kin-Ming Lee, Kwok-Pui Fung, Ping-Chung Leung, Ning-Hua Tan, Clara Bik-San Lau

**Affiliations:** 1 Institute of Chinese Medicine; 2 State Key Laboratory of Phytochemistry and Plant Resources in West China (CUHK); 3 School of Biomedical Sciences, The Chinese University of Hong Kong, Shatin, New Territories, Hong Kong SAR, China; 4 State Key Laboratory of Phytochemistry and Plant Resources in West China, Kunming Institute of Botany, Chinese Academy of Sciences, Kunming 650201, Yunnan, China; 5 University of Chinese Academy of Sciences, Beijing 100049, China

## Abstract

Cancer cells acquire invasive ability to degrade and adhere to extracellular matrix (ECM) and migrate to adjacent tissues. This ultimately results metastasis. Hence, the present study investigated the *in vitro* effects of cyclopeptide glycoside, RA-XII on cell adhesion, invasion, proliferation and matrix degradation, and its underlying mechanism in murine breast tumour cells, 4T1. The effect of RA-XII on tumour growth and metastasis in 4T1-bearing mice was also investigated. Our results showed that RA-XII inhibited tumour cell adhesion to collagen, fibronectin and laminin, RA-XII also reduced the expressions of vascular cell adhesion molecule, intracellular adhesion molecule and integrins, and integrin binding. In addition, RA-XII significantly inhibited breast tumour cell migration via interfering cofilin signaling and chemokine receptors. The activities of matrix metalloproteinase-9 and urokinase-type of plasminogen activator, and the expressions of ECM-associated proteinases were attenuated significantly by RA-XII. Furthermore, RA-XII induced G1 phase arrest and inhibited the expressions of cyclins and cyclin-dependent kinases. RA-XII inhibited the expressions of molecules in PI3K/AKT, NF-kappaB, FAK/pSRC, MAPK and EGFR signaling. RA-XII was also shown to have anti-tumour, anti-angiogenic and anti-metastatic activities in metastatic breast tumour-bearing mice. These findings strongly suggested that RA-XII is a potential anti-metastatic agent for breast cancer.

Metastasis is a leading cause of cancer death. It is responsible for more than 90% breast cancer death^1^. Unfortunately, approximately 20% patients suffering from early-staged breast cancer develop metastasis^2^. Clinically, endocrine therapy, HER2 targeted immunotherapy (such as trastuzmab), chemotherapy (such as doxorubicin, paclitaxel), estrogen receptor modulators (such as tamoxifen) and aromatase inhibitors (such as anastrozole) are commonly used to combat metastatic breast cancer (MBC). However, MBC may be resistant to current conventional chemotherapy, which is always being an obstacle for clinicians. Therefore, a novel anti-metastatic drug is urgently needed.

Activating invasion and metastasis’ is one of the hallmarks of cancer^3^. The mechanisms include, but not limited to, proteolytic enzyme degradation of extracellular matrix (ECM) by cancer cell, cancer cell motility and cancer cell adhesion to the ECM. Suppressing these steps may result in inhibiting metastasis. Cancer cells are able to secrete proteinases such as matrix metalloproteinases (MMPs) to degrade the ECM. MMPs system includes not only MMPs but also urokinase-type plasminogen activator (uPA) and tissue inhibitor of matrix metalloproteinases (TIMPs). Degraded ECM provides a path for cancer cells to migrate as long as they adhere to the ECM. Vascular cellular adhesion molecule (VCAM), intracellular adhesion molecule (ICAM) and integrins expressed on cancer cells are responsible for cell adhesion. Migrating cancer cells at the leading edge adhere to the ECM and recruit actin cytoskeleton and promote membrane protrusion. On the other side, cells at the rear edge detach from the ECM. During cell migration, molecules in cofilin signaling are usually involved. Rho-associated protein kinase 1 (ROCK1) and small G-proteins, RhoA and cell division cycle 42 (CDC42) can stimulate LIM kinase 1 (LIMK1) to phosphorylate cofilin, and thereby attenuate EGF-induced actin nucleation and polymerization, resulting in inhibition of cell migration and invasion^4^. Chemokine receptors can also mediate cancer cell migration preferentially to particular sites where their corresponding ligands are highly expressed. Breast cancer cells highly express CXCR4 and CCR7^5^. Chemokine receptors also regulate cancer cell adhesion through integrin^6^. Integrins can link the ECM to actin cytoskeleton, and mediate cell migration as well as cell adhesion.

Inducing angiogenesis and evading growth suppressors are also the hallmarks of cancer^3^. Suppressing these steps may result in attenuating cancer progression and ultimately inhibiting metastasis. Anti-angiogenic therapy, aimed at suppressing the growth of blood vessels, is a widely accepted strategy to inhibit tumour growth and metastasis. Anti-angiogenic inhibitor, bevacizumab and other drugs with angiogenic activity such as sorafenib (Nexavar*®*), sunitinib (Sutent*®*) have been approved by the U.S. Food and Drug Administration in some cancers^7^. Cancer cells which are capable of evading growth suppressors can proliferate uncontrollable at primary and secondary sites. Cyclins need to be associated with cyclin-dependent kinases (CDKs) to exert their activities in various phases of the cell cycle. Cyclin D1 and CDKs are considered as therapeutic targets for cancer to suppress its growth^8^. Generally speaking, cyclin D-CDK4/6 and cyclin E-CDK2 complexes are involved in G1/S phase, while cyclin A-CDK2 and cyclinB-CDK1 complexes are involved in G2/M phase and mitosis, respectively.

Plant-derived compounds, such as vinblastine, vincristine, irinotecan and paclitaxel can be the sources of anti-cancer agents in clinically^9^. An antitumor bicyclic hexapeptidic glucoside RA-XII, as shown in Fig. 1A, was firstly identified and isolated from *Rubia cordifolia*^10^. RA-XII was also isolated from *Rubia yunnanensis*^11^,^12^. RA-XII could significantly and potently inhibit nitric oxide (NO) overproduction and induction of inducible nitric oxide synthase (iNOS) in LPS-activated murine macrophages^11^. RA-XII not only induced inhibited activities against NO production but also NF-κB activation in activated macrophages. Furthermore, RA-XII was shown to induce potent anti-tumour activities against 11 cancer cell lines including breast cancer cells^12^. However, the anti-tumour and anti-metastatic effects of RA-XII have not been reported.

The present study aimed to investigate the effects of RA-XII on tumour growth and metastasis in metastatic breast tumour bearing mice, and its effects on ECM degradation by MMPs, cell adhesion, cell migration and cell cycle distribution in breast tumour cells. Mouse mammary carcinoma 4T1 cells are highly tumorigenic and highly invasive that can spontaneously metastasize to several distant organs such as lymph nodes, liver, lung and bone. The 4T1 experimental animal model is commonly applied in studying breast cancer metastasis since it is capable of metastasizing spontaneously and efficiently, as well as its metastatic progressive spread is similar to that in human breast cancer^13^. This is the first report to demonstrate the inhibitory effects of RA-XII on tumour growth, angiogenesis and metastasis in mice. RA-XII could also inhibit cell adhesion to ECM proteins, cell motility and migration, proteolytic degradation of the ECM and induce cell cycle arrest in 4T1 cells. The expressions of the molecules involved in these mechanisms were found to be significantly reduced after RA-XII treatment. Furthermore, RA-XII was demonstrated to affect the expressions of proteins in PI3K/AKT, NF-κB, FAK/pSRC, MAPK and EGFR signaling pathways, so as to exert its anti-metastatic effects in breast cancer cells.

## Methods and Materials

### Preparation of RA-XII

**Figure 1:**
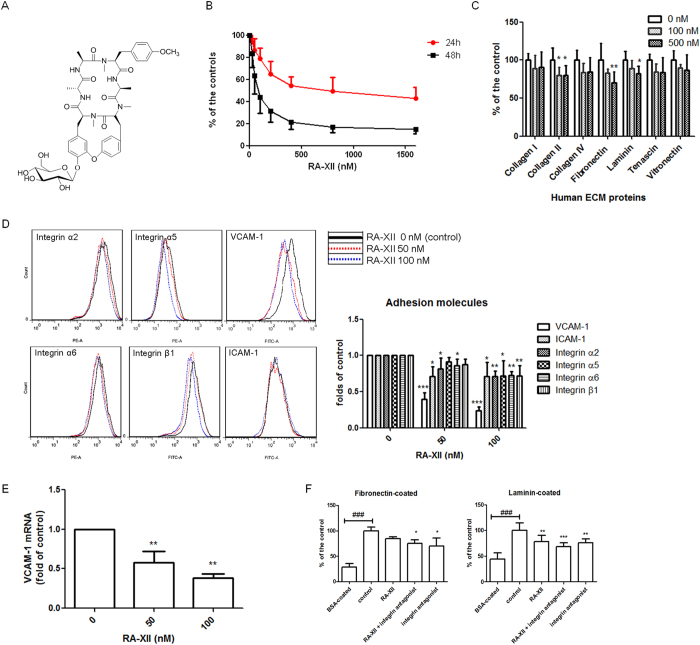
(**A**) Chemical structure of cyclopeptide glucoside RA-XII. (**B**) Cytotoxic effects of RA-XII at 25 to 1600 nM on 4T1cells after 24 or 48 hours by MTT assay. Data were expressed as the mean fold of untreated controls (mean ± SD of 3 independent experiments with 5 replicates each). (**C,D**) Effects of RA-XII on breast cancer cell adhesion. (**C**) In ECM-adhesion assay, 4T1 cells treated with or without RA-XII at 100 or 500 nM were added to wells precoated with ECM proteins for 2 hours. Results were expressed as the mean fold of the untreated controls (mean + SD of 3 independent experiments in duplicates). (**D**) Representative flow cytometric histograms showed the effect of RA-XII (50 or 100 nM treated for 24 hours) on the expressions of adhesion molecules (integrins, VCAM-1 and ICAM-1) on 4T1 cell surface. Cells were stained with FITC- or PE-conjugated antibodies. The histograms on the right panel were expressed as fold of untreated controls (mean + SD of 3-4 independent experiments). (**E**) Effects of RA-XII on VCAM-1 at mRNA levels. 4T1 cells were treated with RA-XII at 50 or 100 nM for 24 hours. The RNA content of cells was isolated and was subjected to real-time PCR. The histograms were expressed as fold of untreated controls (mean + SD of 3 independent experiments with 3 replicates each). (**F**) Effects of RA-XII and/or integrin antagonist on fibronectin- and laminin-cell adhesion assays in 4T1 cells. Cells added to the fibronectin-coated or laminin-coated wells were treated with RA-XII (500 nM) and/or integrin antagonist (10 μM) for 2 hours. BSA-coated wells used as negative control. The intensity of coloured product extracted from the stained bound cells represents cell adhesion to fibronectin or laminin. Results were expressed as the percentage of the control (mean + SD of 3 to 4 independent experiments with 2 replicates each). Statistical differences were determined by One-way ANOVA, followed by Dunnett Test, with *p < 0.05, **p < 0.01, ***p < 0.001 against untreated controls.

RA-XII (Fig. 1A) was isolated from the roots of *Rubia yunnanensis*. The details of extraction and isolation procedures for RA-XII have been reported previously^12^. RA-XII powder was dissolved in dimethyl sulphoxide (DMSO) (Sigma, USA) at 10 mM as a stock solution. The stock was stored at −20 °C and reconstituted in PBS prior to use.

### Preparation of RA-XII nanoemulsion

RA-XII was mixed and stirred with 20 equals of medium chain triglycerides (MCT) and 20 equals of cremophor ELP in a flask. After RA-XII was dissolved, 10 equals of anhydrous ethanol was added to yield the RA-XII nanoemulsion at 15.05 mg/g as stock solution, which was stored at 4 °C. Its particle size is 78.11 nm with the polydispersity index (PDI) at 0.259. For *in vivo* studies, the nanoemulsion was diluted in PBS (1:5, v/v) before use to obtain a working solution of concentration of 3.018 mg/mL, and administered to tumour-bearing mice within 3 hours.

### Cell culture

4T1 mouse mammary carcinoma cells were purchased from American Type Culture Collection (ATCC) and were maintained in RPMI medium 1640 supplemented with 10% (v/v) heat-inactivated fetal bovine serum (FBS) and 100 units/mL penicillin-streptomycin. Primary culture of 4T1 tumour cells was also isolated from 3 tumour-bearing mice and maintained. Tumour cells were allowed to grow until they reached 70% to 80% confluence and subjected for the RA-XII treatment. All the culture media, FBS and supplements were obtained from Life technologies (USA). Cells were incubated at 37 °C in a humidified atmosphere of 5% CO_2_. The cells obtained from ATCC were immediately expanded and frozen down such that all cell lines could be restarted every 3–4 months from a frozen vial of the same batch of cells. Once resuscitated, cell lines were routinely authenticated through cell morphology monitoring and growth curve analysis.

### Cytotoxicity MTT assay

The cytotoxicity of RA-XII on 4T1 was assessed using MTT assay. Cells (3 × 10^4^/mL) were seeded in 96-well flat-bottom culture plate in 100 μL of medium and incubated overnight. RA-XII at various concentrations in 100 μL of medium was added to give final concentrations of 25 to 1600 nM. Medium containing vehicle solvent (0.5% (v/v) DMSO) was added to the wells as the untreated controls. Microplate was then incubated for 24 or 48 hours. Thirty microlitres of 3-(4,5-dimethylthiazol-2-yl)- 2,5-diphenyl tetrazolium bromide (MTT) solution (5 mg/mL) (Sigma, USA) was added and incubated for 3 hours at 37 °C. Supernatant in each well was removed and 100 μL of DMSO was added. Once the violet crystal was dissolved in DMSO, the plate was read at 540 nm by a microplate reader (BioTek, USA). The change in optical density was represented as fold of untreated control.

### Extracellular matrix cell (ECM) adhesion assay

To evaluate the cell adhesion ability of 4T1 cells to human ECM proteins, Extracellular Matrix Cell Adhesion Array Kit (Chemicon, Millipore, USA) was performed. Each well of an eight-well strip was precoated with one of the seven different human ECM proteins (collagen I, collagen II, collagen IV, fibronectin, laminin, tenascin or vitronectin) or bovine serum albumin (BSA), as negative control. The assay was carried out according to the procedures recommended by the manufacturer. Briefly, cells were added to pre-coated wells treated with or without RA-XII at 100 or 500 nM for 2 hours. For fibronectin-cell and laminin-cell adhesion assays, 4T1 cells (3 × 10^5^/mL) were added to the wells precoated with fibronectin (20 μg/mL) or laminin (20 μg/mL) (Sigma, USA). BSA coating was used as negative control. Cells were treated with RA-XII (100 nM), LY294002 (20 μM) (Cell signaling, USA) and/or Gly-Arg-Gly-Asp-Ser (10 μM) (Abcam, USA) for 2 hours. After washing, bound cells were stained and dissolved in extraction buffer. The plate was read at 450 nm by a microplate reader. The change in optical density was represented as fold of untreated control.

### Scratch wound assay

Cell motility of 4T1 cells was assessed using scratch wound assay. Cells (1 × 10^5^/mL) were seeded in a 24-well plate and incubated overnight. The cells were starved with medium in 1% (v/v) FBS for 24 hours. The cells were scraped with a cross by a 200 μL pipette tip in the middle of well. Each well contained 2 crosses. Crosses of each well were photographed under an Olympus IX-71 microscope (Japan) at 0-hour. Fresh medium with 50 or 100 nM of RA-XII were added to the wells in triplicate and the plate was incubated for 24 hours. Crosses of each well were photographed again at 24 hours under microscope. The percentages (%) of open wound area were measured and the change in open wound area (%) at 24-hour against 0-hour was calculated using the Tscratch software^14^. Motility was determined by the decrease of open wound area.

### Transwell migration assay

To assess the cell migration ability of 4T1 cells, a modified Boyden chamber was used in transwell migration assay. Cells (1.5 × 10^5^/mL) in 200 μL of medium with 1% (v/v) FBS containing RA-XII and/or LY294002 (20 μM) at various concentrations were added to the upper chamber of each transwell with 8 μm-pore size filter membrane (Corning, USA) to give final concentration of RA-XII at 50 or 100 nM in duplicate transwells. Five hundred milliliters of medium with 10% (v/v) FBS, as a chemoattractant media, was added to the lower chamber. Transwells were incubated for 4 hours at 37 °C. Cells were then fixed with methanol for 3 minutes and stained with haematoxylin for 5 minutes. Cotton swab was used to scrap the cells on the top surface of the filter membrane of the upper chamber gently. Stained filters were photographed under an Olympus IX-71 microscope (Japan), and stained migrated cells were quantified by manual counting in a double-blinded manner. Migration was determined by the number of migrated cells, and was represented as fold of untreated control.

### Urokinase-type plasminogen activator (uPA) chromogenic activity assay

The activity of uPA of 4T1 cells was assessed using uPA activity kit (Chemicon, Millipore, USA). Cells (5 × 10^4^/mL) were seeded in a 24-well plate overnight. They were then treated with RA-XII at 12.5 to 50 nM in 0.5 mL culture medium with 1% (v/v) FBS and incubated for 48 hours. Conditional medium was collected and centrifuged at 1000 × g for 10 minutes. Supernatants were collected and stored at −20 °C until use. The assay was carried out according to the procedures recommended by the manufacturer. Briefly, conditional medium diluted in assay buffer provided was added to the wells containing uPA substrate and incubated for 24 hours at 37 °C. Secreted uPA from 4T1 cells in the conditional medium would cleave a chromogenic substrate to a coloured uPA-containing sample, and the intensity of the coloured products was measured at 405 nm by a microplate reader (BioTek, USA). The change in optical density was represented as fold of untreated control.

### Gelatin zymography

The activities of MMP-2 and MMP-9 (gelatinases) of 4T1 cells were assessed by gelatin zymography. Cells (5 × 10^4^/mL) were seeded in a 24-well plate overnight. The cells were then treated with RA-XII at 12.5 to 50 nM in 0.5 mL FBS-free culture medium and incubated for 48 hours. Conditional medium was collected and centrifuged at 1000 × g for 10 minutes. Supernatants were subjected to gelatin zymography. The supernatants were resolved by 10% SDS polyacrylamide gel polymerized with 0.1% gelatin. After electrophoresis, the gels were washed with 2.5% Triton X-100 (Sigma, USA) for an hour and incubated in a development buffer (50 mM Tris-HCL, pH 7.5, 150 mM NaCl, 10 mM CaCl_2_) for 20 hours at room temperature. The gels were then stained with 0.25% coomassie blue R-250 (Sigma, USA) for 30 minutes and destained with 10% acetic acid in 5% ethanol until clear zones were observed. The activities of MMP-2 and MMP-9 were observed as the clear zones, digested by MMP-2 and MMP-9 in the conditional medium in the gels, captured by a molecular imager, ChemiDoc XRS+ (Bio-Rad, USA). The clear zones were then quantified using Image J (NIH, USA), and represented as fold of untreated control.

### Western blot analysis

Protein expressions of 4T1 cells were studied by Western blotting. Cells (5 × 10^5^) in 7 mL culture medium were seeded in 100 mm culture dish and incubated overnight. Cells were either treated with RA-XII at 100 nM for various time-points (0, 4, 8, 16, 24 or 48 hours) or treated with or without RA-XII at 50 and 100 nM for 24 hours. After incubation, the attached and floating cells were harvested and washed twice with PBS. Cell pellets were lysed with whole cell extraction buffer (2% SDS, 10% glycerol, 625 mM Tris-HCl, pH 6.8) for 20 minutes with shaking on ice. The samples were then centrifuged at 14,000 × g for 15 minutes at 4 °C. In addition, RA-XII (at 50 or 100 nM) were also treated for 6 hours and EGF (10 ng/mL) for 2 hours prior to cytoplasmic protein extraction for the analysis of EGFR and pEGFR expressions. Cell pellets obtained were subjected to centrifugation at 4 °C. They were lysed with cytoplasmic extraction buffer (Thermo Scientific, USA) for 10 minutes at 4 °C for cytoplasmic cell lysate. The supernatants of whole cell lysate and cytoplasmic cell lysate were collected and stored at −80 °C until use, before subjected to Western blotting. The supernatant proteins were heated at 95 °C for 5 minutes and subsequently resolved by 10% SDS polyacrylamide gel. The proteins in the gel were then transferred to 0.45 mm PVDF membrane (Immobilon, Millipore, USA) at 90 V for 90 minutes. The blots were blocked with 5% non-fat dried milk in Tris-buffered saline Tween 20 (TBST) (20 mM Tris–HCl, pH 7.6, 150 mM NaCl, 0.1%, Tween-20). The blots were incubated with primary antibodies, β-actin (Sigma, USA), MMP2, MMP9, TIMP-1, ROCK1, LIMK1, CDK1, CDK2, CDK4 (Abcam, USA), cyclin A1, cyclin B1, cyclin E1, cyclin D1, CDK6, cofilin, p-cofilin, CDC42, RhoA, TIMP-2, PI3K, AKT, pAKT, FAK, pFAK, pSRC, NF-κB, pNF-κB, IκB, pIκB, ERK1/2, pERK1/2, p38, pp38, JNK, pJNK, and EGFR (Cell Signaling, USA) overnight. These anti-mouse or anti-rabbit primary antibodies were proven by their manufacturers to cross-reactive with mouse antigens. The blots were then washed three times with TBST and were incubated with secondary horseradish peroxidase-conjugated anti-mouse or anti-rabbit antibodies (Life Technologies, USA) for an hour. The signals on the blots were detected using ECL solution (GE Healthcare Life Sciences, Sweden), and were captured by a molecular imager, ChemiDoc XRS+ (Bio-Rad, USA). The signals were quantified using Image J (NIH, USA), and represented as fold of untreated control.

### Flow cytometry analysis

#### Cell surface protein analysis

Protein expressions on 4T1 cell surface were studied by Flow cytometry. The parent tumours cells and the tumour cells from primary culture (5 × 10^5^) in 7 mL culture medium were seeded in 100 mm culture dish and incubated overnight. Cells were treated with RA-XII (50 or 100 nM), LY294002 (Sigma, USA) at 20 μL, BAY11-7082 (Sigma, USA) at 5 μL or untreated for 24 hours. After incubation, the attached and floating cells were harvested and washed twice with PBS. Cells were stained with fluorochrome (FITC or PE)-conjugated monoclonal antibodies, CD47b (integrin α_2_), CD47e (integrin α_5_), CD47f (integrin α_6_), CD29 (integrin β_1_), CD197 (CCR7), CD184 (CXCR4) (MiltenyiBiotec, USA), VCAM-1, ICAM-1 (Abcam, USA) in accordance with the manufacturer’s specifications. These antibodies were proven by their manufacturers to cross-reactive with mouse antigens. Samples were mixed well and incubated at 4 °C for 30 minutes in the dark, and they were washed twice with PBS prior to analysis. The fluorescence of ten thousands events were accumulated and detected by flow cytometer (Canto II, BD Biosciences, USA).

#### Cell cycle analysis

Cell cycle distribution in 4T1 cells was assessed by propidium iodide (PI) staining. Cells (1 × 10^5^/mL) were seeded in a 6-well plate overnight. After overnight incubation, medium was replaced with FBS-free medium and cells were again incubated overnight. The cells were then treated with RA-XII at 50 or 100 nM in 10% FBS in culture medium and incubated for 24 hours. Attached cells were harvested, washed twice with PBS and fixed in 70% (v/v) ethanol at 4 °C overnight. Cells were resuspended in 500 μL of PBS containing 20 μg/mL propidium iodide (Sigma, USA) and 10 μg/mL RNase A (Sigma, USA) at 37 °C in the dark for 30 minutes. Cell cycle phase distribution was determined by flow cytometry. The fluorescence of ten thousands events were accumulated and detected by flow cytometer (Canto II, BD Biosciences, USA).

### [Methyl-^3^H]-thymidine incorporation assay

The effect of RA-XII on tumour cell proliferation was assessed by [methyl-^3^H]-thymidine incorporation assay. Cells (5 × 10^4^/mL) in 100 μL were seeded in 96-well flat-bottom culture plate and incubated for 24 hours. RA-XII at 100 and 200 nM (100 μL) was added to give final concentrations of 50 and 100 nM. Microplate was incubated for 24 hours. Fifty microlitres of [methyl-^3^H]-thymidine solution (0.5 μCi) (PerkinElmer, USA) was added and incubated for another 6 hours at 37 °C. Plates were subsequently freezed at −20 °C for 24 hours. Following thawed, plates were subjected to scintillation counter (Packard, USA) measurement. The change in count per minutes was represented as fold of untreated control.

### Real-time PCR analysis

The RNA level of proteins in 4T1 cells were analyzed by real-time PCR. Cells (5 × 10^5^) in 7 mL culture medium were seeded in 100 mm culture dish and incubated overnight. Cells were treated with or without RA-XII (50 or 100 nM) for 24 hours. Total RNA was isolated from cells using Trizol reagents according to the procedures recommended by the manufacturer. The RNA concentration was spectrophotometrically determined using a BioPhotometer (Eppendor). Each PCR samples contained 80 ng cDNA. Reactions were performed in triplicate by Bio-Rad CFX96^TM^ Real-time system C1000 Thermal cycler using the iTaq Fast SYBR Green Supermix from Bio-Rad. The specific gene mRNA levels were normalized relative to GAPDH mRNA level in each sample.

### *In vivo* study

Female Balb/c mice (6 to 8 weeks old) were provided and maintained by Laboratory Animal Services Centre, the Chinese University of Hong Kong. One hundred microliters of 4T1 cells (3 × 10^6^/mL) were inoculated into the mammary fat-pad of the mice on day 0. From day 16, mice were injected intravenously with RA-XII at 5, 10 or 20 mg/kg or PBS (control) twice a week. After 6 treatments, mice were sacrificed on day 40. Blood, tumours, livers and lungs were collected for further analysis. All experimental methods in mice were carried out in accordance with the approved guidelines and regulations specified by the Animal Experimentation Ethics Committee of the Chinese University of Hong Kong (CUHK). All experimental protocols were approved by the Animal Experimentation Ethics Committee of CUHK with reference numbers Ref No. 10/051/MIS.

### Plasma enzyme analysis

Blood collected from the mice was centrifuged at 4000 rpm for 10 minutes. Plasma was collected and stored at −80 °C prior to use. The levels of plasma enzymes, aspartate aminotransferase (AST), alanine aminotransferase (ALT), alkaline phosphatase (ALP) and creatine kinase (CK) were measured using quantitative determination of enzyme kits (Stanbio Laboratory, USA). The assay was carried out according to the procedures recommended by the manufacturer.

### Deparaffinization and hydration of embedded tissues

Tumours or organs were fixed in 10% formalin (Sigma, USA) for 3 days prior to sample embedding. Paraffin sections of embedded tissues (tumours, liver or lung) on the slides (5 μm) were incubated in a dry oven at 60 °C for 30 minutes. Tissues were dewaxed by xylene for 2 minutes for 3 times with shaking, and were hydrated gradually in 100% ethanol twice, then 95%, 80% and 70% ethanol for 2 minutes each with shaking. They were then washed with running water for 5 minutes.

### Haematoxylin and eosin staining

The slides with lung or liver tissues were immersed in haematoxylin for 5 minutes, and then immediately immersed with acidic alcohol. Subsequently, they were immersed with bluing agent (Thermo Scientific, USA) for 2 minutes, followed by immersed with eosin (Thermo Scientific, USA) for 30 seconds. Washing with tap water for 30 seconds was required between each step described above. They were then immersed with 75% ethanol for 30 seconds. Subsequently, they were dehydrated gradually in 90%, 100% ethanol twice for 2 minutes, followed by immersed with 100% xylene for 2 minutes for 3 times. Several sections at 2 levels (500 μm between each level) of each liver tissue and lung tissue were photographed (x100) and tumour area was measured in a blinded-manner.

### Immunohistochemistry in primary tumour tissues

The slides with tumour tissues were immersed in peroxidase I blocking reagent (Biocare, Medical, USA) for 5 minutes and immersed in preheated Diva Decloaker (Biocare Medical, USA). They were heated in a microwave for 20 minutes. The tissues were then treated with rat anti-CD31 IgG2a (Dianova, Germany) at 4 °C overnight. After washing with PBS for 1 minute for 3 times, a rat-on-mouse-HRP-polymer kit (Biocare Medical, USA) containing rat-on-mouse-HRP-polymer and rat probe was applied according to the procedures recommended by the manufacturer. After washing the slides with PBS for 1 minute for 3 times, pre-warmed 3,3′-Diaminobenzidine (DAB) (Open Biosystems, USA) were added onto the tissues for 5 minutes at 55 °C, and the slides were then immersed with haematoxylin (Thermo Scientific, USA) for 30 seconds. After washing with tap water, they were immersed with 70% and 80% ethanol quickly with shaking. They were then dehydrated gradually in 90%, 100% ethanol twice for 2 minutes, followed by immersed with 100% xylene for 2 minutes for 3 times. The expression of CD31 was visualized on the sections, appeared as brown in colour. Several sections at 2 levels (500 μm between each level) of each tumour tissue were photographed (x100), and the number of CD31 expressed cells was counted per sections in a blinded-manner.

### Statistical analysis

Data were expressed as mean + SD for *in vitro* study and mean + SEM for *in vivo* study. Statistical analyses and significance were calculated by One-way ANOVA, followed by Dunnett test and analyzed using GraphPad PRISM software version 5.0 (GraphPad Software, USA), to compare the groups with the control. Statistical difference was also determined by unpaired Student’s *t*-test to compare between two groups. In all comparisons, p < 0.05 was considered as statistically significant.

## Results

### RA-XII induced cytotoxicity in breast tumour cells

The cytotoxicity of RA-XII on 4T1 cells for 24 and 48 hours were determined by MTT assay (Fig. 1B). The IC_50_ values (the concentrations producing 50% cell death) of RA-XII on 4T1 cells were 606 nM and 96 nM for 24 and 48 hours, respectively. Since our approach was to investigate the *in vitro* anti-metastatic effects of RA-XII on cancer cell adhesion, migration and invasion without inducing high cytotoxicity, low concentrations of RA-XII were applied in the culture assays. Hence, RA-XII at up to 100 nM was applied in the following culture assays for 24-hour treatment; and up to 50 nM for 48-hour treatment.

### RA-XII reduced breast tumour cell adhesion to ECM proteins

The ability of tumour cells to adhere to matrix proteins was assessed by ECM adhesion assay. Figure 1C showed the reduced adhesion to matrix proteins in 4T1 cells treated with RA-XII for 2 hours. RA-XII at 100 and 500 nM could significantly decrease cell adhesion to collagen II to 80% of the untreated controls, and at 500 nM significantly reduced cell adhesion to fibronectin and laminin to 70% and 82% of the controls, respectively. RA-XII could also slightly inhibit its adhesion to fibronectin and laminin at 100 nM.

### RA-XII inhibited the expressions of integrins and adhesion molecules, VCAM and ICAMs, and integrin binding in breast tumour cells

Integrins and adhesion molecules, VCAM and ICAMs are involved in cell-cell or cell-matrix adhesion. Results from flow cytometry (Fig. 1D) revealed that RA-XII at 50 and 100 nM significantly and dose-dependently reduced VCAM-1, ICAM-1 and integrin subunits α_2_, α_6_ and β_1_ expressions on 4T1 cells, while at 100 nM, RA-XII significantly inhibited integrin α_5_ level when treated for 24 hours. RA-XII at 100 nM even dramatically reduced VCAM-1 expression to 23% of the controls. Since RA-XII could reduce VCAM-1 expression to very low level, the effect of RA-XII on VCAM-1 at mRNA level was investigated. VCAM-1 at mRNA level was also greatly reduced to 39% of the controls when treated with RA-XII at 100 nM for 24 hours (Fig. 1E).

As RA-XII reduced matrix-cell adhesion and the expressions of integrins in 4T1 cells, it was suggested that RA-XII reduced matrix-cell adhesion via reducing integrin binding. Gly-Arg-Gly-Asp-Ser is an integrin antagonist, as a sequence (Arg-Gly-Asp) provides an attachment site for integrins. Integrin antagonist (10 μM) alone and RA-XII (500 nM) plus integrin antagonist reduced 4T1 cell adhesion to fibronectin to the similar extent i.e. 70% of the control in fibronectin-cell adhesion assay (Fig. 1F). This result demonstrated that the anti-adhesive effect of RA-XII to fibronectin was via reducing its integrin binding. In contrast, RA-XII and integrin antagonist inhibited cell adhesion to 78% and 76%, respectively in laminin-cell adhesion assay (Fig. 1F). The combined RA-XII plus integrin antagonist treatment reduced cell adhesion to 69% (p < 0.001), which was only slightly more than single treatment, revealing that RA-XII reduced laminin-cell adhesion in 4T1 cells partially via reducing integrin binding.

### RA-XII inhibited breast tumour cell motility and migration

**Figure 2:**
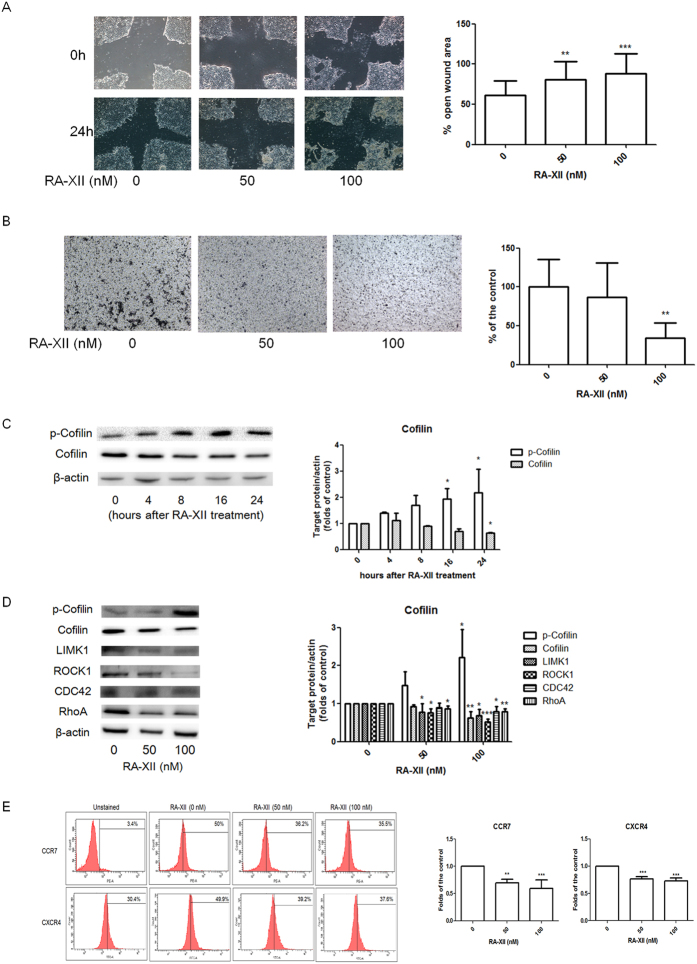
Effects of RA-XII on 4T1 cell motility and migration. (**A**) Cells were treated with or without RA-XII at 50 and 100 nM for 24 hours in scratch wound assay. Representative photographs showed the effects of RA-XII at 50 and 100 nM on cancer cell motility, and quantified analysis summarized the percentage of the change in open wound area at 24-hour compared with those at 0 h (mean + SD of 3 independent experiments with 6 replicates each). (**B**) Cells were treated with or without RA-XII at 50 or 100 nM for 4 hours in transwell migration assay. Representative photographs showed the stained migrated cancer cells on the lower side of the membrane after incubation. Quantified analysis summarized the number of migrated cells on the lower chambers (mean + SD of 3 independent experiments with duplicates each) and expressed as the percentage of the control. (**C**) Representative western blots showed the effect of RA-XII at 100 nM on cofilin and p-cofilin expressions at various time-points. (**D**) Representative western blots revealed the effect of RA-XII at different doses after 24-hour treatment. The cropped blots have been obtained under the same experimental conditions and the full-length blots were shown in Supplementary information. The histograms in **(C**,**D**) showed the expressions of the target proteins which were normalized with corresponding β-actin protein expression and expressed as fold of control (mean + SD of 3-4 independent experiments). (**E**) Representative flow cytometric histograms showed the inhibitory effect of RA-XII (50 or 100 nM treated for 24 hours) on the expressions of chemokine receptor, CCR7 on 4T1 cell surface. Cells were stained with PE-conjugated CCR7 antibodies. The histograms were expressed as fold of untreated controls (mean + SD of 3-4 independent experiments). Statistical differences were determined by One-way ANOVA, followed by Dunnett Test, with *p < 0.05, **p < 0.01, ***p < 0.001 against untreated controls.

In scratch wound assay, Fig. 2A showed the cells of untreated controls have been migrated towards each other, with decreasing open wound area, while the motility of RA-XII-treated breast tumour cells was inhibited in scratch wound assay. The histograms showed that the open wound area (%) of the cells treated with RA-XII at 50 and100 nM was significantly greater than that of untreated control, indicating that RA-XII at 50 and 100 nM significantly inhibited 4T1 cell motility in a dose-dependent manner. In transwell migration assay, cells have been migrated across the pore of the membrane from the upper chamber of the transwell to the lower chamber. The histograms showed that the number of migrated cells was significantly decreased when cells were treated with RA-XII at 100 nM (Fig. 2B), revealing that RA-XII could inhibit 4T1 cell migration.

### RA-XII inhibited the expressions of the molecules involved in cofilin signaling

Cofilin signaling is involved in actin polymerization as well as cell migration. Phosphorylated cofilin (inactivated form of cofilin) and cofilin are the key molecules involved in cofilin signaling, so the effects of RA-XII at 100 nM on these molecules in 4T1 cells at different time-points were investigated. Western immunoblot showed that RA-XII increased p-cofilin expression and decreased cofilin expression from 4 hour to 24 hour. Quantified results revealed that RA-XII significantly increased p-cofilin from 16- to 24-hour, and reduced cofilin expression at 24-hour (Fig. 2C). Therefore, the effects of RA-XII on various molecules in cofilin signaling were determined at 24-hour after treatment. Significantly reduced cofilin and increased p-cofilin expressions were found in 4T1 cells treated with RA-XII at 100 nM (Fig. 2D). RA-XII at 50 to 100 nM also significantly inhibited the expressions of RhoA, ROCK1, LIMK1 in dose-dependent manner while significantly inhibited CDC42 expression at 100 nM.

### RA-XII reduced CCR7 and CXCR4 expressions in breast tumour cells

Chemokines can direct the migration of cancer cells expressing corresponding chemokine receptors from primary site to specific organ. In Fig. 2E, RA-XII at 50 to 100 nM could significantly reduce the expressions of CCR7 in 4T1 cells in a dose-dependent manner. However, negligible level of CXCR4 was found from parent 4T1 cell line (data not shown). Primary culture cells were therefore isolated from 4T1 tumours as they express CXCR4 in tumour microenvironment. RA-XII at 50 to 100 nM significantly inhibited the CXCR4 expression in the primary culture extracted from 4T1 tumours from 3 mice (p < 0.001).

### RA-XII attenuated TIMPs and MMPs expressions and reduced MMP-9 and uPA activities in breast tumour cells

**Figure 3:**
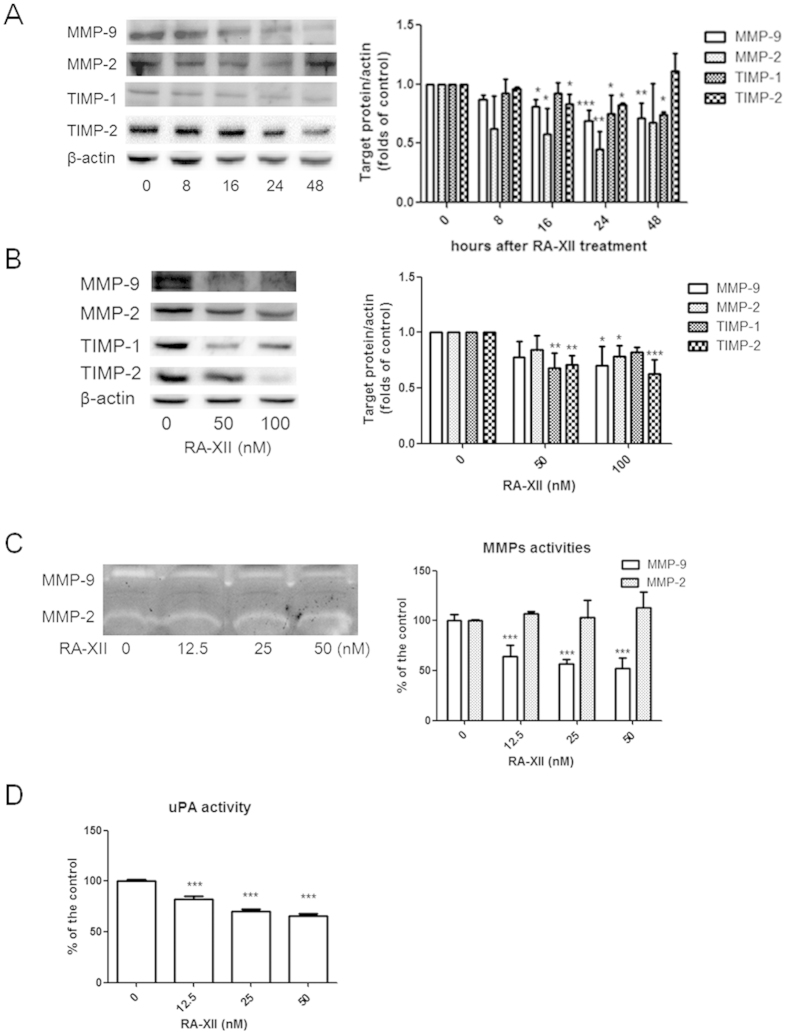
Effects of RA-XII on matrix degradation. Representative western blots showed the effect of RA-XII on the expressions of ECM-associated proteinases, MMP-2, MMP-9, TIMP-1 and TIMP-2 in 4T1 cells (**A**) at various time-points with 100 nM of RA-XII, and (**B**) at different doses for 24 hours. The cropped blots have been obtained under the same experimental conditions and the full-length blots were shown in Supplementary information. The histograms showed the quantified results of the expressions of the ECM-associated proteinases, which were normalized with corresponding β-actin protein expression and expressed as fold of untreated controls (mean + SD of 3 independent experiments). (**C,D**) 4T1 cells were treated with or without RA-XII at 12.5 to 50 nM for 48 hours, and conditional medium were subjected to (**C**) gelatin zymography and (**D**) uPA activity assay. In gelatin zymography, representative photographs showed the activities of MMP-2 and MMP-9 on the stained gelatin gel. The effects on MMPs activities were represented by the digested gelatin on the gel in uPA chromogenic activity assay, uPA activity was measured using a specific uPA substrate releasing a coloured chromophore. Data were expressed as fold of untreated controls (mean + SD of 3 independent experiments with duplicates each). Statistical differences were determined by One-way ANOVA, followed by Dunnett Test, with *p < 0.05, **p < 0.01, ***p < 0.001 against untreated controls.

TIMP-1 and TIMP-2 are MMP inhibitors for MMP-9 and MMP-2, respectively. The time-dependent and dose-dependent effects of RA-XII on the expressions of the ECM-associated proteinases, MMP-2, MMP-9, TIMP-1 and TIMP-2 were studied in 4T1 cells using western blot. RA-XII at 100 nM time-dependently inhibited the expressions of MMP-2 and TIMP-2 from 16 to 24 hours; MMP-9 from 16 to 48 hours; TIMP-1 from 24 to 48 hours (Fig. 3A). Since the expressions of the above proteinases were significantly decreased at 24-hour, the dose-dependent effect of RA-XII on their expressions was studied at 24-hour. RA-XII at 100 nM could significantly inhibit MMP-2, MMP-9 and TIMP-2 expressions, and at 50 nM could significantly reduce TIMP-1 expression at 24-hour (Fig. 3B).

Gelatinases (MMP-9 and MMP-2) and uPA (plasminogen activator) are ECM-associated proteinases involved in matrix degradation. The activity of gelatinases and uPA can be investigated using gelatin zymography and uPA chromogenic activity assay, respectively. The activities of MMP-9 and uPA secreted by 4T1 cells were significantly inhibited by RA-XII at 12.5 to 50 nM in a dose-dependent manner for 48 hours (Fig. 3C,D).

### RA-XII inhibited cell proliferation and induced G1 arrest in breast tumour cells

**Figure 4:**
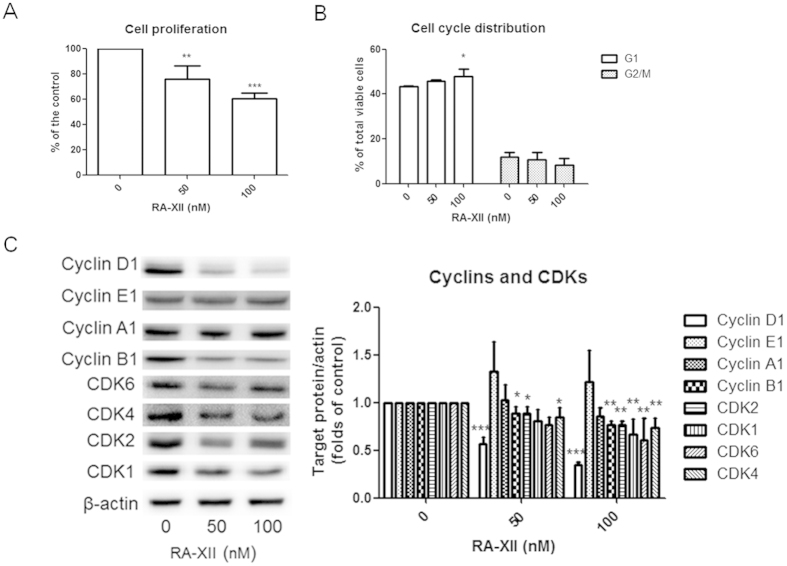
Effects of RA-XII on cell proliferation. (**A**) Effect of RA-XII on cell proliferation was investigated by [methyl-^3^H]-thymidine incorporation assay. 4T1 tumour cells were treated with RA-XII (50 and 100 nM) for 18 hours. They were subsequently incubated with [methyl-^3^H]-thymidine for 6 hours. Cell proliferation was revealed by the amount of [methyl-^3^H]-thymidine uptaken by cells, and expressed as the percentage of the control (mean + SD of 3 independent experiments). (**B**) Effect of RA-XII on cell cycle distribution. 4T1 tumour cells, treated with RA-XII (50 and 100 nM) for 24 hours, were analyzed by flow cytometry. Bar charts summarized the percentages of total viable 4T1 cells in G1 and G2/M phases (mean + SD of 3–4 independent experiments). (**C**) Representative western blot showed the effects on the expression of cyclins and CDKs when 4T1 cells were treated with RA-XII at 50 and 100 nM for 24 hours. The cropped blots have been obtained under the same experimental conditions and the full-length blots were shown in Supplementary information. The histograms summarized the expressions of the target proteins which were normalized with corresponding β-actin protein expression and expressed as fold of untreated controls (mean + SD of 3–4 independent experiments). Statistical differences were determined by One-way ANOVA, followed by Dunnett Test, with *p < 0.05, **p < 0.01, ***p < 0.001 against untreated controls.

RA-XII significantly reduced cell proliferation to 76% at 50 nM and 61% at 100 nM by [methyl-^3^H]-thymidine incorporation assay (Fig. 4A). In order to further investigate the effect of RA-XII on cell cycle distribution, propidium iodide-stained cells were analyzed by flow cytometry. After 24-hour treatment of RA-XII at 100 nM, the number of viable cells undergoing G1 phase in the cell cycle were significantly and dose-dependently increased (Fig. 4B), suggesting that RA-XII could induce G1 arrest in breast tumor cells. RA-XII could also induce G1 arrest in time-dependent manner as the number of cells undergoing G1 phase after 24-hour treatment was increased compared with 16-hour treatment (data not shown).

To investigate whether RA-XII was capable of inhibiting the productions of cyclins and CDKs involved in cell cycle progression, western blotting experiments were carried out. Cyclin D1 and CDK4/6 are involved in G1/S transition. RA-XII (100 nM) significantly reduced cyclin D1 and CDK4/6 expressions in 4T1 cells treated for 24 hours (Figure C). This might be associated with G1 phase arrest induced by RA-XII. The expressions of cyclin B1 and CDK1, involved in mitosis, were also reduced in RA-XII-treated 4T1 cells. RA-XII (50 and 100 nM) also reduced CDK2 expressions in dose-dependent manner. The expressions of cyclin E1 and cyclin A1, involved in late G1 phase and G2/M phase respectively, were not significantly affected by RA-XII.

### RA-XII interfered FAK/pSRC, NF-κB, PI3K/AKT, MAPK and EGFR signaling in breast tumour cells

**Figure 5:**
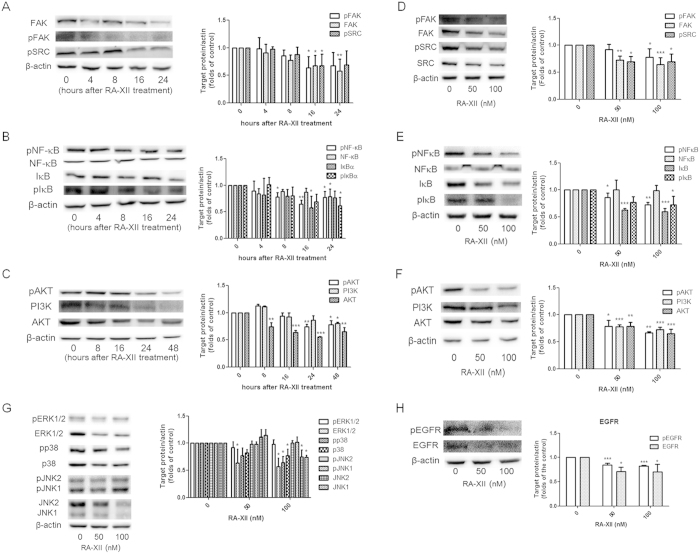
Effects of RA-XII on the expressions of molecules in various signaling pathways in breast cancer cells. Representative blots revealed the expressions of the signaling molecules involved in (**A**) FAK/pSRC, (**B**) NF-κB/IκB and (**C**) PI3K/AKT in 4T1 cells treated with RA-XII at 100 nM at various time-points. Representative blots also showed the expressions of the signaling molecules involved in (**D**) FAK/pSRC, (**E**) NF-κB/IκB, (**F**) PI3K/AKT and (**G**) MAPK signaling pathways in 4T1 cells treated with RA-XII at 50 and 100 nM for 24 hours. Whole cell lysate was subjected to Western blotting. (**H**) Representative blots showed the expressions of the signaling molecules involved in EGFR signaling in 4T1 cells treated with RA-XII at 50 and 100 nM for 6 hours and EGF (10 ng/mL) for 2 hours, prior to cytoplasmic protein extraction. The cropped blots have been obtained under the same experimental conditions and the full-length blots were shown in Supplementary information. The histograms showed the quantified results of the expressions of the target proteins involved in various pathways which were normalized with corresponding β-actin protein expression and expressed as folds of control (mean + SD of 3–4 independent experiments). Statistical differences were determined by One-way ANOVA, followed by Dunnett Test, with *p < 0.05, **p < 0.01, ***p < 0.001 against untreated controls.

As our previous findings demonstrated the anti-metastatic effects of RA-XII on 4T1 cells, the effect of RA-XII on different signaling pathways was further investigated. The effects of RA-XII at 100 nM on FAK/pSRC, NF-κB and PI3K/AKT signaling pathways were evaluated at various time-points. RA-XII significantly reduced the expressions of FAK, pFAK and pSRC at 16-hour (Fig. 5A). RA-XII also significantly inhibited the phosphorylation of NF-κB at 8 to 24 hours (Fig. 5B). At 24 hours, pNF-κB level was slightly increased at 24-hour compared to 16-hour. In addition, RA-XII interfered PI3K/AKT signaling (Fig. 5C).The level of AKT in 4T1 cells was significantly decreased since 8-hour treatment, while pAKT and PI3K expression were not significantly reduced until 24 and 48 hours after RA-XII treatment, respectively.

RA-XII (50 and 100 nM) dose-dependently reduced the signaling molecules in FAK/pSRC, NF-κB and PI3K/AKT signaling pathways in 4T1 cells treated for 24 hours. Figure 5D showed that RA-XII (100 nM) significantly inhibited pFAK, FAK and pSRC expressions in 4T1 tumour cells. RA-XII (50 and 100 nM) also signficantly inhibited the phosphorylation of SRC in dose-dependent manner. In addition, RA-XII (50 and 100 nM) significantly and dose-dependently inhibited the phosphorylation of NF-κB while the level of NF-κB was unaffected (Fig. 5E). For PI3K/AKT signaling, RA-XII significantly and dose-dependently reduced AKT and PI3K levels and inhibited the phosphorylation of AKT (Fig. 5F). For MAPK signaling, RA-XII at 100 nM significantly attenuated the expressions of ERK1/2, JNK1/2 and p38 while only inhibited the phosphorylation of p38 (Fig. 5G). In addition, RA-XII (50 and 100 nM) could inhibit the phosphorylation and expression of EGFR in 4T1 tumour cells treated for 6 hours, in the presence of EGF (10 ng/mL) (Fig. 5H).

### RA-XII exerted its anti-invasive activities partly via PI3K/AKT signaling

**Figure 6:**
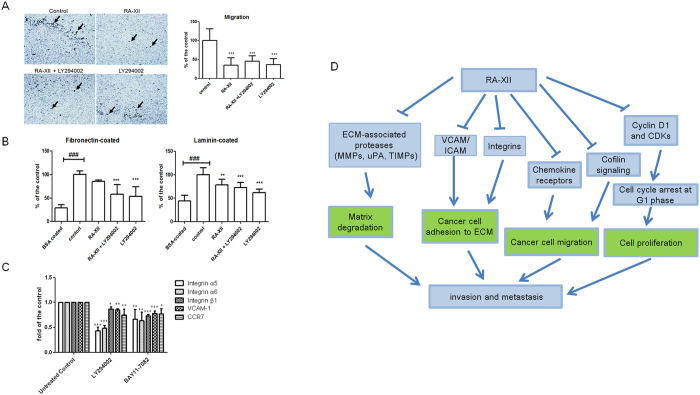
Effects of cell migration and matrix-cell adhesion via PI3K/AKT signaling in 4T1 tumour cells. (**A**) In transwell migration assay, cells were treated with or without RA-XII (100 nM), LY294002 (PI3K inhibitor) (10 μM) and RA-XII plus LY294002 for 4 hours. Representative microscopic photographs showed the stained migrated cells on the lower side of the membrane. Quantified analysis summarized the number of the migrated cells of the membrane and expressed as the percentage of the control (mean + SD of 3 independent experiments with duplicate each). (**B**) In fibronectin-cell and laminin-cell adhesion assays, cells were treated with or without RA-XII (100 nM), LY294002 (PI3K inhibitor) (10 μM) and RA-XII plus LY294002 for 2 hours. Cells added to the fibronectin-coated or laminin-coated wells were treated with or without RA-XII (500 nM), LY294002 (PI3K inhibitor) (10 μM) and RA-XII plus LY294002 for 2 hours. BSA coating was used as the negative control. The intensity of the coloured product from the stained bound cells determines their adhesions to fibronectin or laminin. Results were expressed as the percentage of the control (mean + SD of 3 independent experiments with duplicates each). (**C**) Effects of LY294002 (PI3K inhibitor), BAY11-7082 (NF-κB inhibitor) and PF-573228 (FAK inhibitor) on the expressions of the molecules involved in cell migration and adhesion in breast cancer cells. Cells were treated or without LY294002 at 20 μM, BAY11-7082 at 5 μM and PF-573228 at 1 μM for 24 hours. Cells were stained with FITC- or PE-conjugated antibodies. The histograms summarized the flow cytometric analysis from 3 to 4 independent experiments and were expressed as fold of the controls (mean + SD). Statistical differences were determined by One-way ANOVA, followed by Dunnett Test, with *p < 0.05, **p < 0.01, ***p < 0.001 against the controls. Statistical differences were also determined by unpaired Student’s *t*-test, with ^###^p < 0.001 between 2 groups. (**D**) Schematic diagram of the proposed underlying mechanisms of the anti-metastatic effect of RA-XII.

As RA-XII reduced cell invasion as well as inhibit the phosphorylation and expression of PI3K and AKT in 4T1 cells, the inhibitory effects of RA-XII on cell migration and matrix-cell adhesion via PI3K/AKT signaling was investigated. Therefore, the effects of RA-XII, LY294002 (PI3K inhibitor) and RA-XII plus LY294002 on cell migration and adhesion were determined. In transwell migration assay (Fig. 6A) and fibronectin-cell and laminin-cell adhesion assays (Fig. 6B), the inhibitory effects on tumour cell migration fibronectin-cell adhesion and laminin-cell adhesion between LY294002-treated group and RA-XII-plus-LY294002-treated group were more or less the same. This suggested that RA-XII induced its anti-migratory effect and anti-adhesive effect to laminin and fibronectin via PI3K signaling. In addition, whether the molecules involved in cell adhesion and cell migration are the downstream molecules of PI3K/AKT and NF-κB signaling were also investigated. Figure 6C showed that both LY294002 (PI3K inhibitor) at 20 μM and BAY11-7082 (NF-κB inhibitor) at 5 μM significantly reduced the expressions of integrins α_5_, α_6_ and β_1_, VCAM-1 and CCR7. LY294002 and BAY11-7082 even reduced integrins α_5_ and α_6_ by more than 50% (p < 0.001) and 40% (p < 0.01), respectively. These findings strongly suggested that RA-XII could reduce the expressions of these molecules via PI3K/AKT and NF-κB signaling so as to reduce 4T1 cell invasion. On the other hand, PF-573228 (FAK inhibitor) at 1 μM did not significantly reduce the expressions of these molecules, suggesting that the inhibitory effect of RA-XII on expressions of adhesion molecules and chemokine receptor was not via FAK/pSRC signaling.

### The RA-XII intravenous treatment induced no toxicity in breast tumour-bearing mice

**Figure 7:**
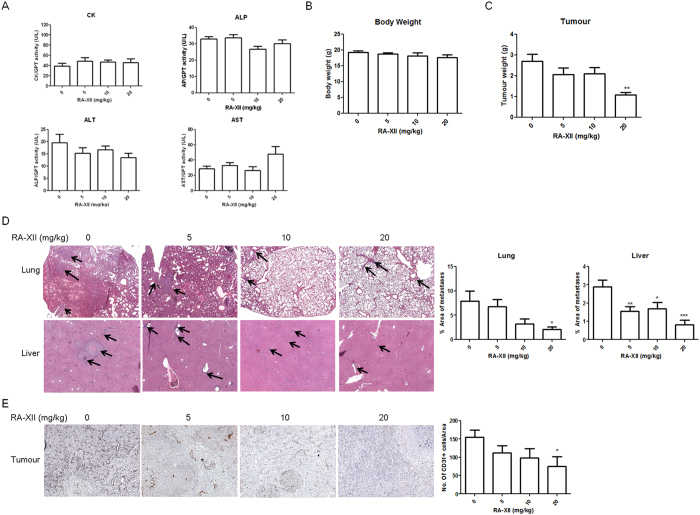
Effects of RA-XII on tumour growth and metastasis in a 4T1-tumour bearing mice model. Mice were treated with RA-XII at 5, 10 or 20 mg/kg intravenously twice a week for 3 weeks. (**A**) The levels of serum enzymes, aspartate aminotransferase (AST), alanine transaminase (ALT), alkaline phosphatase (ALP) and creatine kinase (CK) of blood plasma collected from the mice in each group. (**B**) Body weight and (**C**) tumour weight of the mice at the end of experiment. Representative photographs of (**D**) haematoxylin and eosin staining of lungs and livers tissues with arrows showing the tumours, and (**E**) immunohistochemistry against CD31 in tumour tissues. The histograms summarized the quantified data expressed as mean + SEM of 5 to 7 mice from each group. Statistical differences were determined by One-way ANOVA, followed by Dunnett Test, with *p < 0.05, **p < 0.01, ***p < 0.001 against untreated controls.

There was no significant difference in the levels of plasma enzymes, aspartate aminotransferase (AST), alanine transaminase (ALT), alkaline phosphatase (ALP) and creatine kinase (CK) among the mice from each group (Fig. 7A), suggesting that no significant damage to liver or heart was induced by RA-XII intravenous treatments. In addition, there was no significant difference in the body weight of mice between treatment and control groups (Fig. 7B), indicating that RA-XII treatment had no severe toxicity effect to the treated mice. After all courses of RA-XII treatment, only one mouse treated with 10 mg/kg was died.

### RA-XII inhibited breast tumour metastasis to lung and liver

Tissues of liver and lung from the mice were subjected to haematoxylin and eosin staining to study the distribution of tumour cells to secondary organs. Figure 7D showed the representative photographs of 4T1 cells metastasized to the lungs and livers from the mice in each group. Area of tumour nodules in the lungs was significantly reduced when mice were treated with 20 mg/kg of RA-XII, while area of metastases in the livers was significantly reduced when treated with RA-XII at 5 to 20 mg/kg (Fig. 7D). This suggested that RA-XII at 20 mg/kg could inhibit breast cancer metastasis to both liver and lung.

### RA-XII inhibited primary tumour growth and angiogenesis

The tumour weight of the mice treated with RA-XII at 20 mg/kg was significantly reduced, as shown in Fig. 7C, demonstrating the inhibitory effect of RA-XII on tumour growth. Since new blood vessels formed allows the tumours to grow, the effect of RA-XII on angiogenesis in primary tumours was further studied. CD31 is a representative marker of endothelial cells, forming the inner lining of blood vessels. CD31 positive cells in tumours from the mice treated with RA-XII at 20 mg/kg were significantly reduced (Fig. 7E), revealing the inhibitory effect of RA-XII on angiogenesis in tumours. The tumour weight was decreasing with the number of blood vessel, suggesting that inhibiting angiogenesis in tumour might be the mechanism of RA-XII to inhibit tumour growth.

## Discussion

Cancer cells need to acquire invasive abilities so as to migrate through basal membrane to the surrounding circulation. Some of these invasive cancer cells are capable of surviving in the circulation and arresting and colonizing in a secondary site^15^. The above mechanisms require modified cancer cell adhesion, cell migration, cell proliferation, and ECM proteolysis. Our *in vitro* results revealed that RA-XII reduced the invasive potential of 4T1 cells such as reducing cell migration, cell adhesion, cell cycle arrest and expressions and activities of proteolytic enzymes involved in matrix degradation. Figure 6D clearly outlined a schematic diagram of the proposed underlying mechanisms of the anti-metastatic effect of RA-XII, based on our *in vitro* results. Our *in vivo* results further confirmed that RA-XII could inhibit breast tumour metastasis to liver and lung in tumour bearing mice. Angiogenesis in tumours also allow primary tumours or secondary tumours to grow even larger. Suppressing these processes can lead to attenuated tumour growth and metastasis. Our study showed that RA-XII could inhibit the metastatic tumour growth *in vivo*, and this may be associated with the inhibitory effects of RA-XII on angiogenesis in primary tumours.

Cancer cell-ECM interaction plays a critical role in invasion and metastasis, and allows signal transduction across the membrane. Our ECM adhesion assay data revealed that RA-XII could significantly reduce the adhesion of 4T1 cells to collagen II, fibronectin and laminin which are the major components of the ECM. RA-XII also inhibited the expressions of α_2_, α_5_, α_6_ and β_1_. As α_2_β_1_, α_5_β_1_ and α_6_β_1_ are involved in cell adhesion to collagen, fibronectin and laminin, respectively^16^, the reduced expressions of α_2_β_1_, α_5_β_1_ and α_6_β_1_ are likely contributed to the anti-adhesive effect of RA-XII on ECM-cell adhesion. In addition, adhesion molecules on cancer cells play multiple roles in metastasis rather than only facilitate cell adhesion. For example, integrins regulate cell adhesion, migration, proliferation, invasion, angiogenesis and metastasis^17^,^18^. A study revealed that α_v_β_3_ antagonist suppressed both cell migration and adhesion to vitronectin, while α_5_β_1_ antagonist suppressed cell migration on vitronectin without affecting cell adhesion, suggesting that integrins could directly interfere signaling pathway resulting in cell migration^17^. Failure in adhering to ECM is also resulted in cell cycle arrest via reducing cyclin D1 mRNA level, and inactivating cyclin E-CDK2^19^. Besides, RA-XII reduced 4T1 cell adhesion via ICAM-1 and VCAM-1 downregulation. VCAM-1 was identified as one of the 18 genes mostly associated with breast cancer metastasis to lung^20^, and VCAM-1- or integrin α_4_-blocking antibodies could inhibit breast cancer metastasis to bone *in vivo*^21^. It is generally accepted that VCAM-1 facilitates cancer cell metastasis via enhancing adhesive ability of cancer cells. Surprisingly, VCAM-1 facilitated lung colonization through providing survival signaling to breast cancer cells^22^. Blocking integrin inhibited not only cell adhesion but also cell invasion and MMP-9 activity in breast cancer^23^. These studies revealed the multiple roles of VCAM-1 and integrins in cancer metastasis.

As mentioned, cancer cells need to become invasive so as to metastasize. Cancer cells with invasive ability allow themselves to migrate to adjacent tissues, followed by intravasation into circulation and extravasation to secondary site. RA-XII significantly inhibited 4T1 cell motility and migration, as shown in scratch wound assay and transwell migration assay, respectively. This may be associated with the inhibitory effects of RA-XII on the expressions of cofilin and LIMK1 in cofilin signaling. Cofilin induces actin polymerization and directs cell migration. A review summarized that increased cofilin, decreased phosphorylated cofilin and increased LIMK1 expressions were usually found in highly invasive cancer cells^24^. High level of LIMK1 was responsible for tumour angiogenesis and breast cancer metastasis *in vivo*^25^. However, in contrast, overexpressed LIMK1 was also found to reduce cancer cell motility^26^. It is because LIMK1 phosphorylates cofilin so as to inactivate it, resulting in reduced cell motility. This comes up with a question that the inhibitory effect of RA-XII on LIMK1 expression may promote cell migration. In fact, a study demonstrated that both up-regulated cofilin and LIMK1 expressions were identified in invasive cancer cells isolated from the primary mammary tumours^24^. This coordinated overexpression of cofilin and LIMK1 might increase the rate of cofilin acticity in invasive cancer cells, leading to actin polymerization transients as well as facilitating cancer cell invasion. Therefore, a balance between LIMK1 and cofilin levels is suggested to determine migration and invasion in tumour cells.

Accumulating evidences suggested that breast cancer cells highly express active CCR7 and CXCR4 which are responsible for directing breast cancer cell migration and invasion, and it potentially leads to metastasis to liver, lung, bone marrow and lymph nodes. Constitutive expression of CXCL12, specific ligand for CXCR4, was usually found in several organs such as liver, lung, bone marrow, while CCL21 and CCL19, specific ligands for CCR7, are highly expressed in lymph node^5^. CCR7 expression was found to be associated with lymph node metastasis^27^. RA-XII inhibited CCR7 expression on 4T1 cells, and downregulating CCR7 level might be one of the mechanisms involved in inhibiting metastasis in RA-XII-treated 4T1 tumour-bearing mice. However, our results showed that negligible level of CXCR4 was detected on 4T1 cells but high level of CXCR4 was found from the primary culture of 4T1 tumours *in vivo*. Our observation was consistent with some studies which demonstrated that CXCR4 did express in gene level *in vitro* or when isolated from the primary tumours *in vivo*. However, the lack of CXCR4 expression was found in murine colon carcinoma cells *in vitro*. These findings suggested the requirement of *in vivo* tumour microenvironment to upregulate CXCR4 expression^28^. Upon binding to chemokine receptors expressed on cancer cells, chemokine is able to regulate the reorganization of actin cytoskeleton via intracellular signaling. Activation of chemokine receptors can also induce actin polymerization and mediate migration, adhesion as well as integrin activation^29^. These may be responsible for the inhibitory effects of RA-XII on cell adhesion as well as cell migration and motility.

When migrating cancer cells adhere to ECM, degraded ECM provides a path for invasive cancer cells during metastasis. Therefore, inhibiting the expression and activity of ECM-associated proteinases are responsible for inhibiting matrix degradation and invasion. MMP-9 is associated with poor prognosis of patients suffering from breast cancer^30^. High level of uPA is associated with reduced relapse-free survival and overall survival^31^. Our results demonstrated that MMP-9 and uPA activities in 4T1 cells were reduced when treated with RA-XII for at least 48 hours, suggesting that RA-XII is able to inhibit ECM degradation and hence cancer cell invasion. Being MMP inhibitors, TIMPs are expected to inhibit cancer progression, invasion and metastasis^32^. In contrast, RA-XII was shown to decrease TIMP-1 and TIMP-2 expressions in 4T1 cells. Such controversy may be due to the multifunction of TIMP-1 and TIMP-2 such as promoting cell proliferation and angiogenesis^33^. A balance between MMPs and TIMPs determine ECM degradation and synthesis, and an imbalance between MMPs and TIMPs can predict poor prognosis^34^. Such balance is usually associated with the ratio of MMPs expression to TIMPs expression. Although RA-XII could significantly reduce TIMP-1 expression at 50 nM but not 100 nM (Fig. 3B), RA-XII at 50 and 100 nM could significantly inhibit the ratio of MMP-9 to TIMP-1 expressions in a dose-dependent manner (data not shown). Reduced activity and protein expressions of ECM-associated proteinases in 4T1 cells by RA-XII may therefore result in reduced matrix degradation and cancer cell invasion. Although RA-XII could reduce MMP-2 expression (Fig. 3B), it could not attenuate MMP-2 activity in 4T1 cells (Fig. 3C). This may due to the reduced expression of a MMP-2 inhibitor, TIMP-2 by RA-XII.

Uncontrolled cancer cell proliferation can promote primary and secondary tumour growth. RA-XII inhibited cell proliferation in 4T1 cells. Overexpressed cyclin D1 was found in 50 to 70% breast cancers while CCND1 amplification, cyclin D1 gene was found in 15 to 20% breast cancer^35^. Cyclin D1 and CDKs play a role in regulating the G1 phase of the cell cycle. RA-XII was found to induce G1 phase arrest, and this might be associated with reduced cyclin D1 and CDKs expressions by RA-XII. Phosphorylated retinoblastoma protein (Rb) induced by cyclin D1-CDK4/6 complex results in the expressions of genes involved in cell cycle progression such as cyclin E via E2F-regulated transcription. This suggested that reduced cyclin D1 expression is usually followed by reduced cyclin E expression. However, our result showed that RA-XII significantly reduced cyclin D expression but slightly up-regulated cyclin E expression. Sandor *et al.* (2000) investigated the effects of a cyclic peptide inhibitor of histone deacetylase and found the same contradictory findings and suggested that it was due to the inhibitory effects on histone deacetylase^36^. Since reduced cyclin D level can lead to dephosphorylated Rb bound E2F complexes, these complexes recruit histone deacetylase and exert its active transcriptional repression^37^. Therefore, inhibiting histone deacetylase might prevent the downregulation of cyclin E and hence increased cyclin E expression. RA-XII could also reduce cyclin B1 and CDK1 in which cyclin B1-CDK1 complex is involved in mitosis. By inducing cell cycle arrest, RA-XII could inhibit cancer cell proliferation so as to inhibit the growth of primary tumour and secondary tumours in liver and lung in tumour-bearing mice.

RA-XII was shown to inhibit PI3K/AKT, FAK/pSRC, NF-κB, MAPK p38 and EGFR activations, so it might exert its anti-metastatic effects via these signaling pathways. Although our early study has indicated that RA-XII could reduce PI3K activity and FAK signaling pathway^38^, there was no full-detailed methodology and experimental results included in the patent application. RA-XII could inhibit the expression of molecules in FAK/pSRC signaling in which they were responsible for breast cancer cell adhesion, migration and invasion^39^. Reduced breast cancer cell migration^40^ and activity of ECM-associated proteinases involved in matrix degradation^41^ could be achieved by blocking PI3K/AKT and NF-κB signaling pathways. LY294002 (PI3K inhibitor) could inhibit cancer cell proliferation and induce G1 arrest, accompanied by reduced cyclin D1 and CDK4 levels^42^. In addition, activating PI3K/AKT/IKKβ/NF-κB signaling can result cyclin D1 induction^43^. These suggested that RA-XII likely inhibited cyclin D1 levels through PI3K/AKT signaling. Our data (Fig. 6) also showed that some adhesion molecules (integrins and VCAM-1) and chemokine receptor (CCR7) are the downstream signaling molecules of PI3K and NF-κB signaling. PI3K/AKT signaling can also be triggered by integrins^44^. In addition, RA-XII could affect the expression of proteins involved in PI3K and NF-κB signaling, and RA-XII could inhibit expression of integrins, VCAM and CCR7. These findings strongly suggested that RA-XII inhibited breast cancer invasion and metastasis via PI3K/AKT and NF-κB signaling pathways.

RA-XII is not only a potential anti-metastatic agent but also a potential anti-tumour agent. Our *in vivo* study demonstrated the inhibitory effects of RA-XII on tumour growth and metastasis in breast tumour-bearing mice. Furthermore, RA-XII inhibited angiogenesis in tumours with reduced tumour growth. This finding strongly suggested that RA-XII is a potential anti-angiogenic agent, and its ability to inhibit angiogenesis might be responsible for its anti-tumour activity.

This is the first study demonstrating in details that RA-XII could interfere PI3K/AKT, FAK/pSRC, MAPK and EGFR signaling pathways so as to inhibit breast cancer cell invasion *in vitro* and RA-XII inhibited tumour growth and metastasis *in vivo*. Our *in vitro* study suggested the inhibitory effect of RA-XII on cofilin signaling molecules, chemokine receptors, adhesion molecules (VCAM and ICAM), integrins, MMPs, cyclins and CDKs, as these molecules are strongly associated with each other in metastasis. Our *in vivo* study further confirmed the anti-metastatic potential of RA-XII to inhibit metastasis to lung and liver in metastatic breast tumour-bearing mice. RA-XII also reduced primary tumour growth, associated with attenuated angiogenesis in primary tumour *in vivo*. In summary, RA-XII inhibited breast tumour growth and metastasis *in vivo*, associated with the anti-metastatic potential of RA-XII to inhibit breast cancer cell adhesion, cell migration, cell proliferation and ECM degradation. Therefore, RA-XII is potentially an anti-metastatic as well as anti-tumour agent.

## Additional Information

**How to cite this article**: Leung, H.-W. *et al.* RA-XII inhibits tumour growth and metastasis in breast tumour-bearing mice via reducing cell adhesion and invasion and promoting matrix degradation. *Sci. Rep.* **5**, 16985; doi: 10.1038/srep16985 (2015).

## Supplementary Material

Supplementary Information
