# Supplementary material for: RA-XII inhibits tumour growth and metastasis in breast tumour-bearing mice via reducing cell adhesion and invasion and promoting matrix degradation
Source: Sci Rep. 2015 Nov 23;5:16985. doi: 10.1038/srep16985 (PMC4655310; doi:10.1038/srep16985)

Supplementary Information

Full length blots of the blots shown in manuscript

RA-XII inhibits tumour growth and metastasis in breast tumour-bearing mice via reducing cell adhesion and invasion and promoting matrix degradation

Hoi-Wing Leung[1,2,#], Si-Meng Zhao[4,5,#], Grace Gar-Lee Yue[1,2], Julia Kin-Ming Lee[1,2], Kwok-Pui Fung[1,2,3], Ping-Chung Leung[1,2], Ning-Hua Tan[4,*]and Clara Bik-San Lau[1,2,*]

[1]Institute of Chinese Medicine; [2]State Key Laboratory of Phytochemistry and Plant Resources in West China (CUHK), [3]School of Biomedical Sciences, The Chinese University of Hong Kong, Shatin, New Territories, Hong Kong SAR, China. [4]State Key Laboratory of Phytochemistry and Plant Resources in West China, Kunming Institute of Botany, Chinese Academy of Sciences, Kunming 650201, Yunnan, China. [5] University of Chinese Academy of Sciences, Beijing 100049, China.

Running title: RA-XII inhibits growth and metastasis in breast tumours

* Corresponding authors

Clara Bik-San Lau

E-mail: claralau@cuhk.edu.hk

Tel: (852) 3943 6109

Fax: (852) 2603 5248

Ning-Hua Tan

E-mail: nhtan@mail.kib.ac.cn

Tel: (86) 871 6522 3800

Fax: (86) 871 6522 3800

# Equal contribution

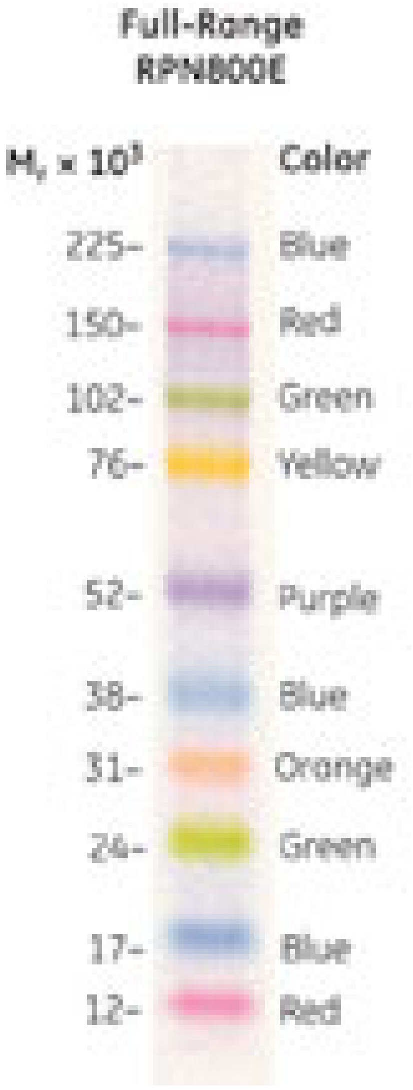

Amersham Full-range rainbow molecular weight marker was used for our western blots

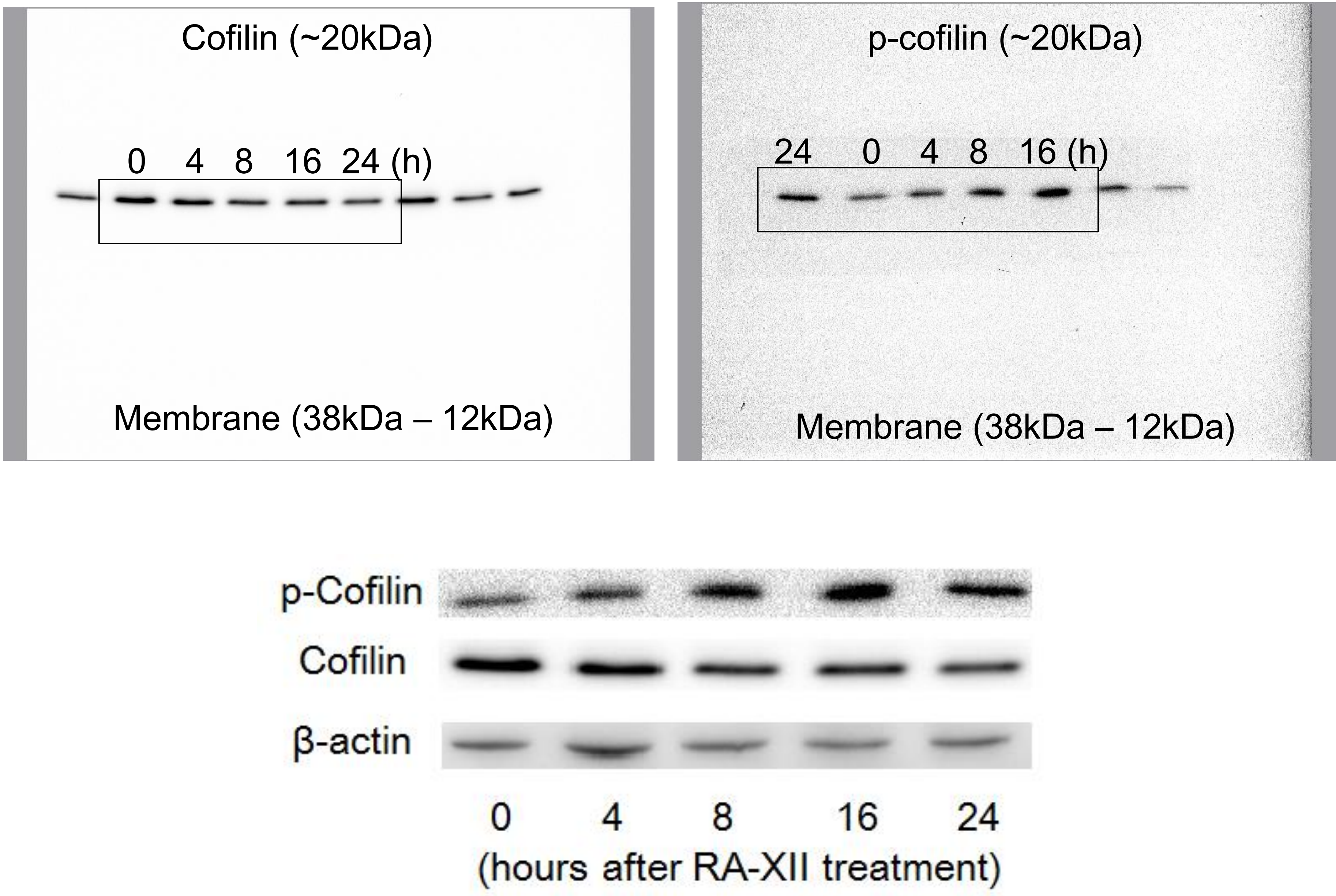

Immunoblots from Figure 2

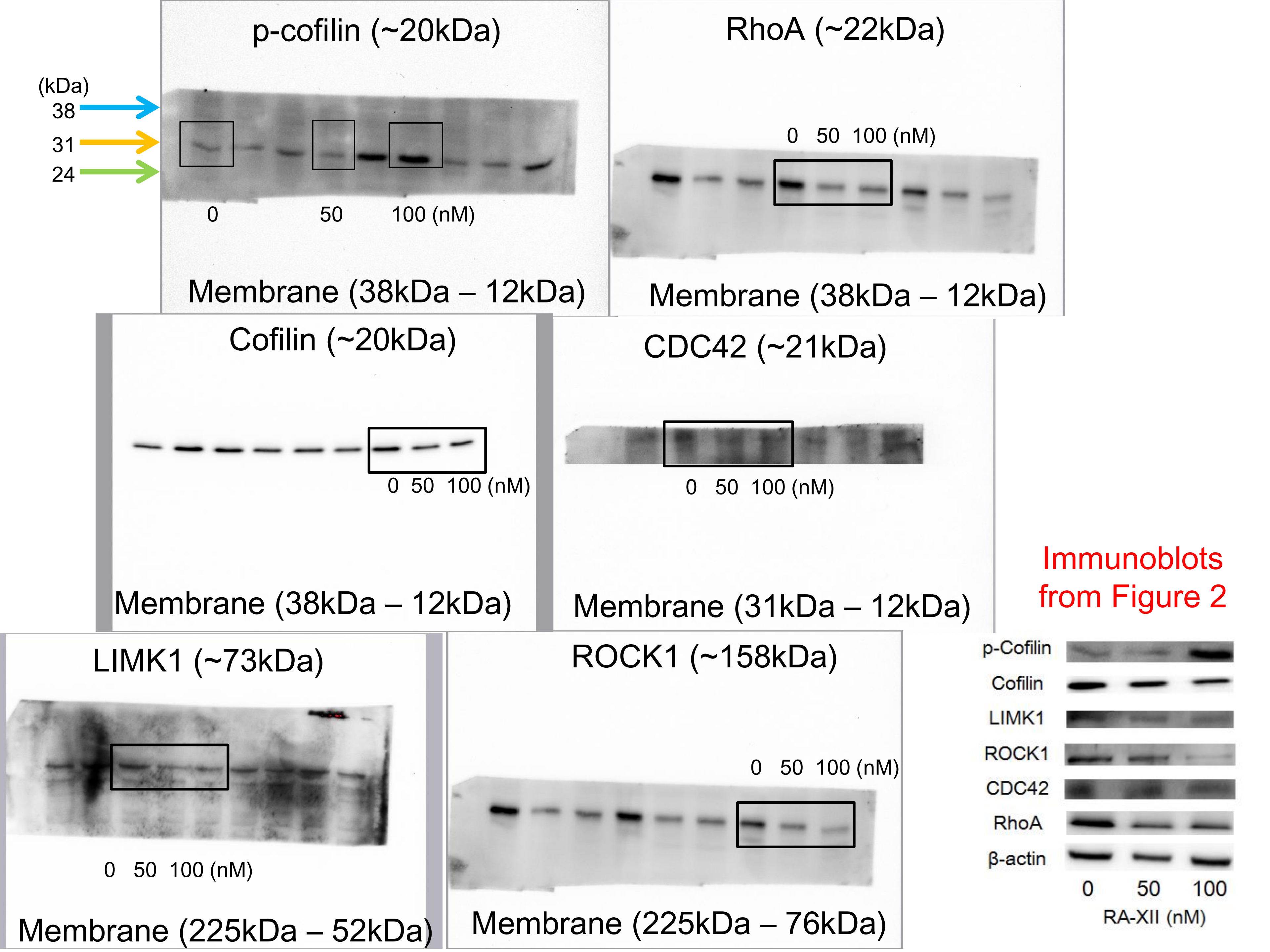
p-cofilin (~20kDa)
(kDa)
38
31
24
0
50
100 (nM)
Membrane (38kDa – 12kDa)
RhoA (~22kDa)
0 50 100 (nM)
Membrane (38kDa – 12kDa)
Cofilin (~20kDa)
0 50 100 (nM)
Membrane (38kDa – 12kDa)
CDC42 (~21kDa)
0 50 100 (nM)
Membrane (31kDa – 12kDa)
Immunoblots from Figure 2
LIMK1 (~73kDa)
0 50 100 (nM)
Membrane (225kDa – 52kDa)
ROCK1 (~158kDa)
0 50 100 (nM)
Membrane (225kDa – 76kDa)
p-Cofilin
Cofilin
LIMK1
ROCK1
CDC42
RhoA
β-actin
0 50 100
RA-XII (nM)

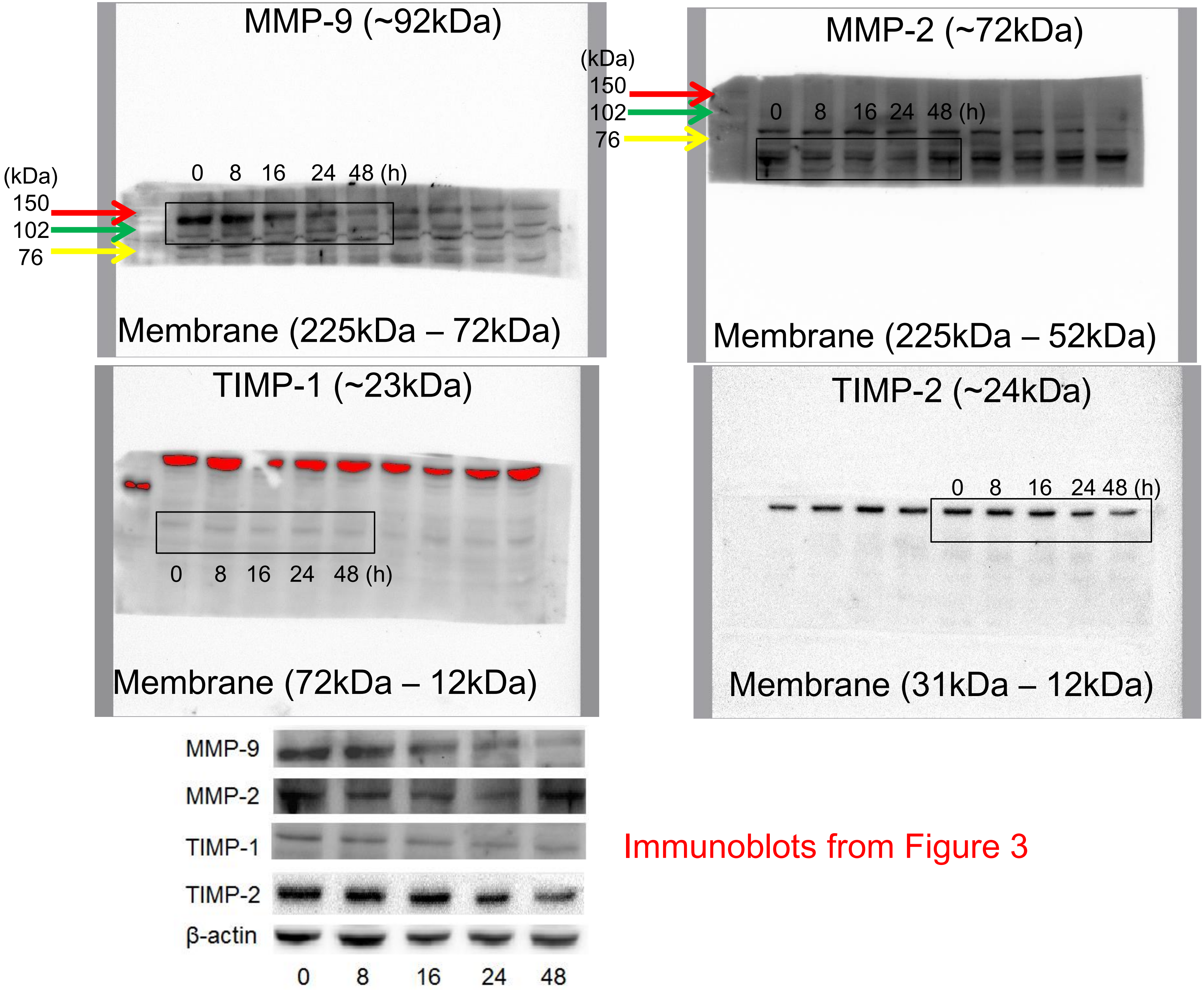

Immunoblots from Figure 3

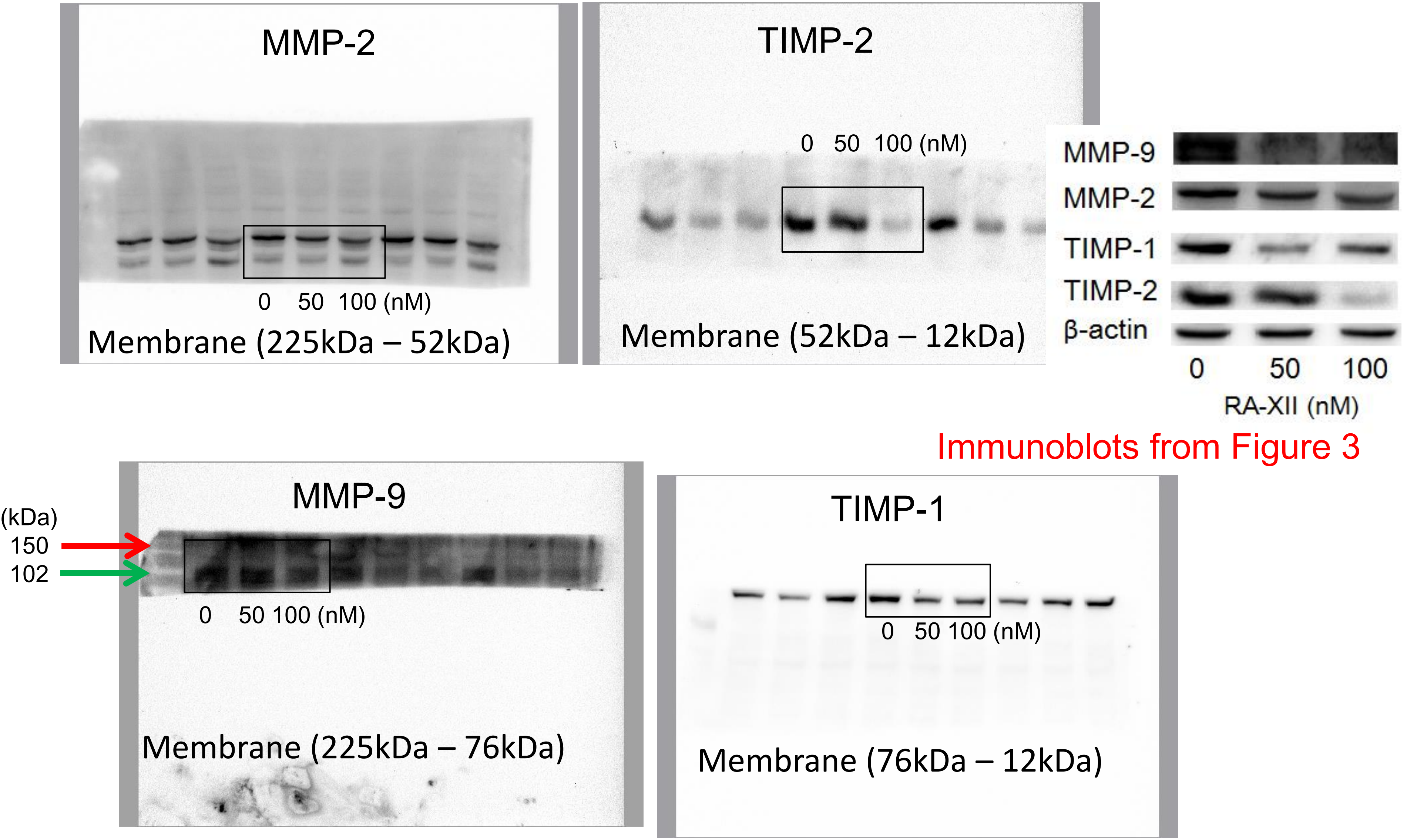

Immunoblots from Figure 3

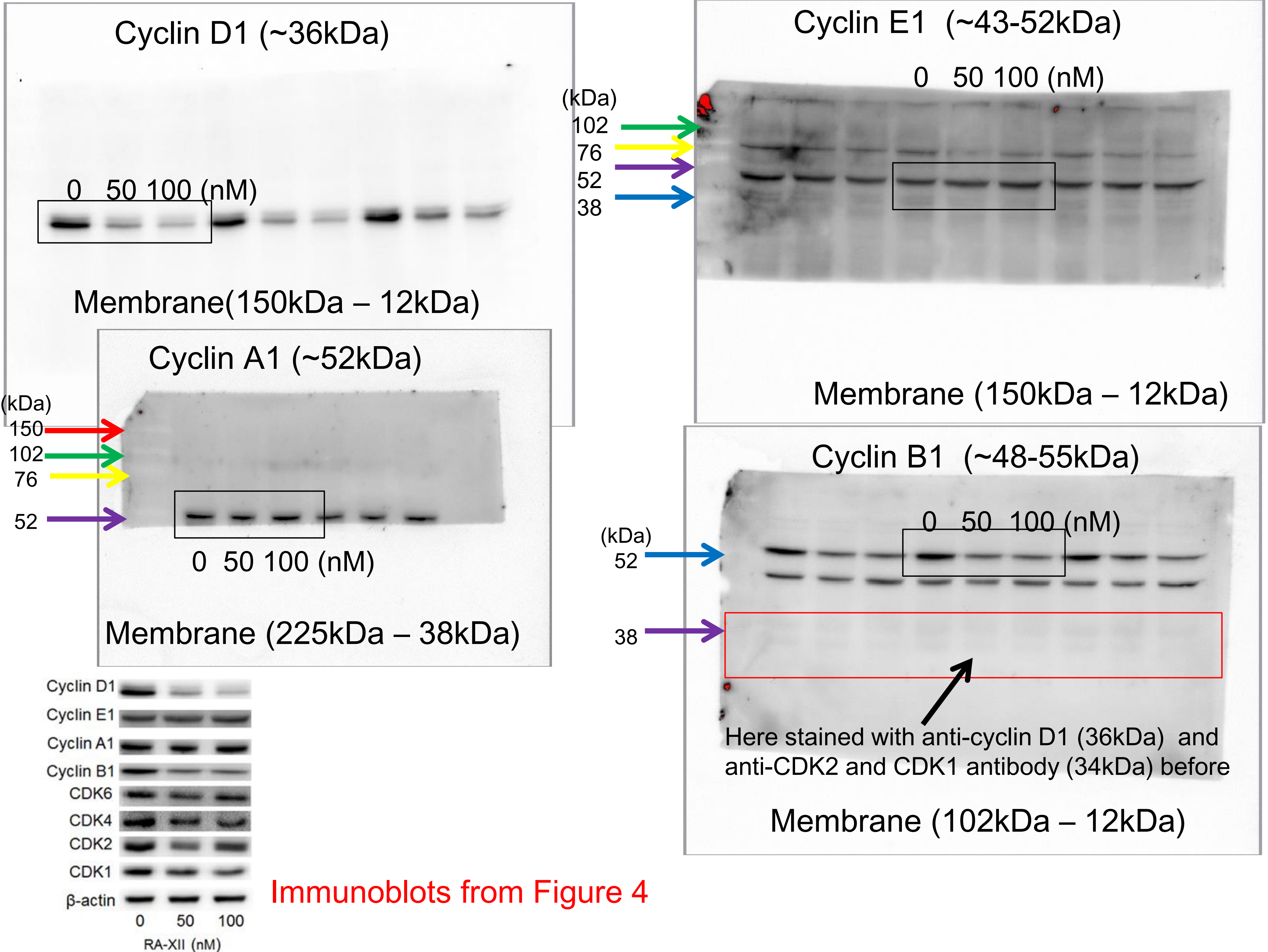

Immunoblots from Figure 4

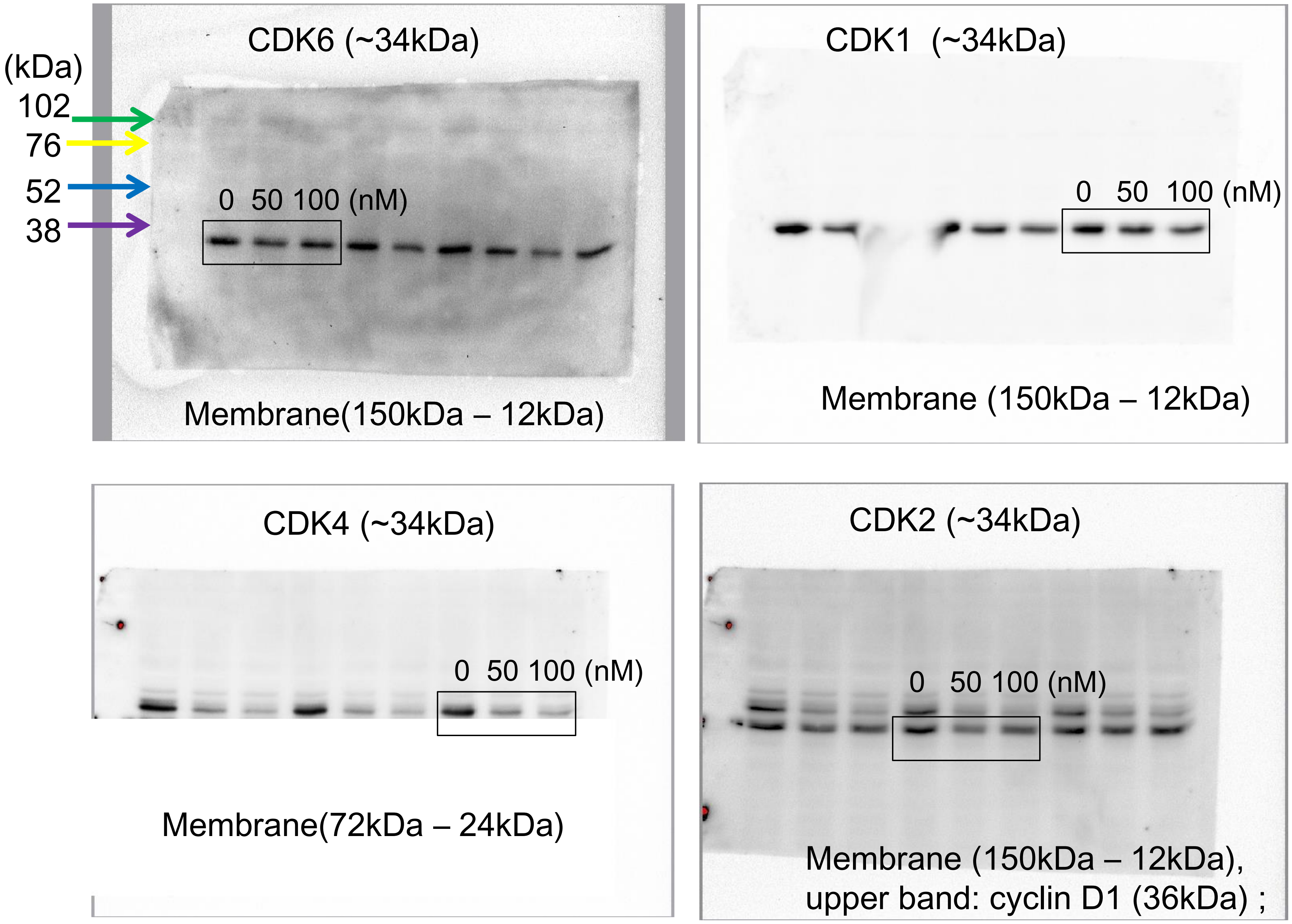

Immunoblots from Figure 4

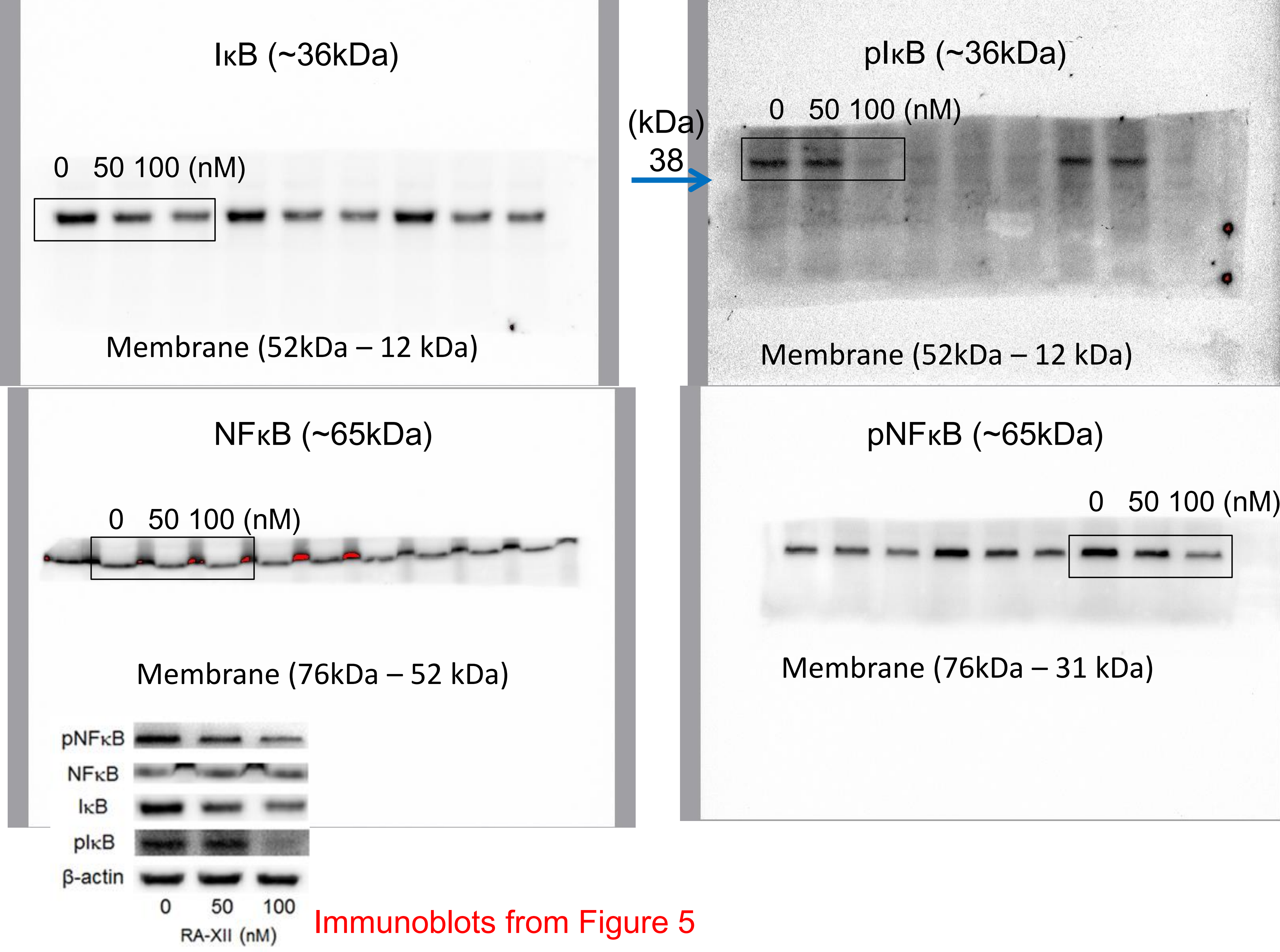

Immunoblots from Figure 5

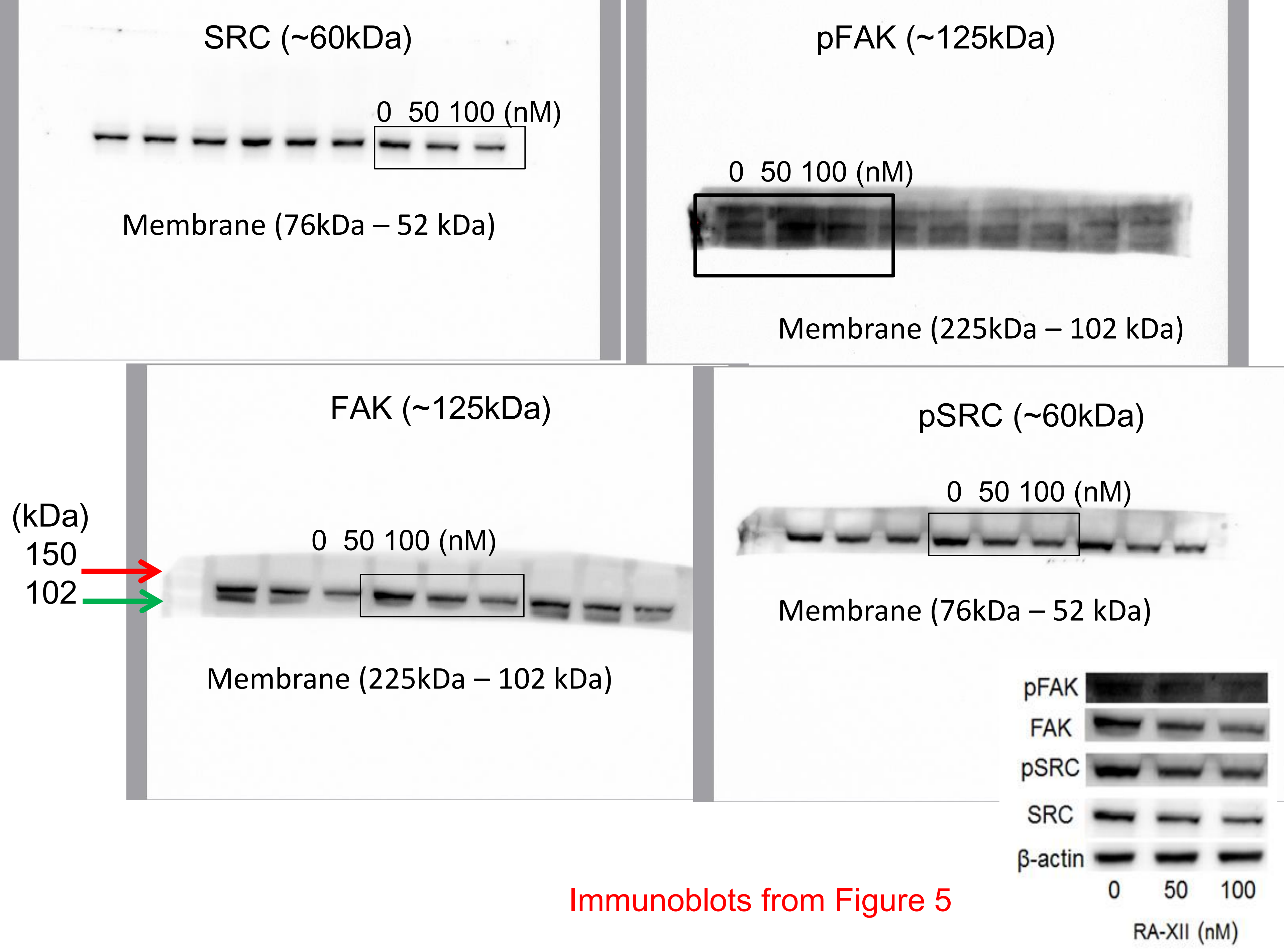

Immunoblots from Figure 5

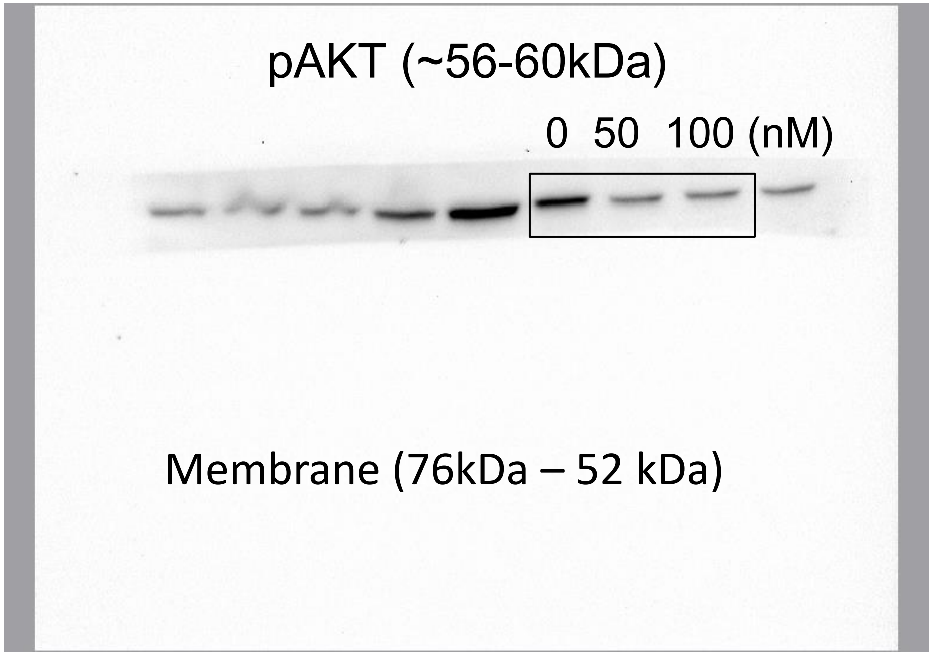

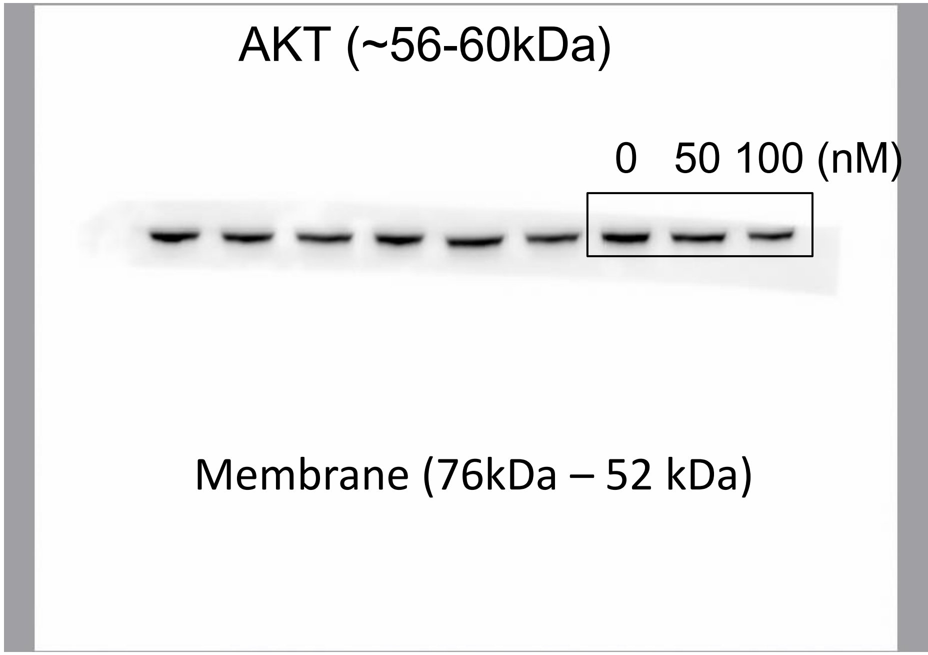

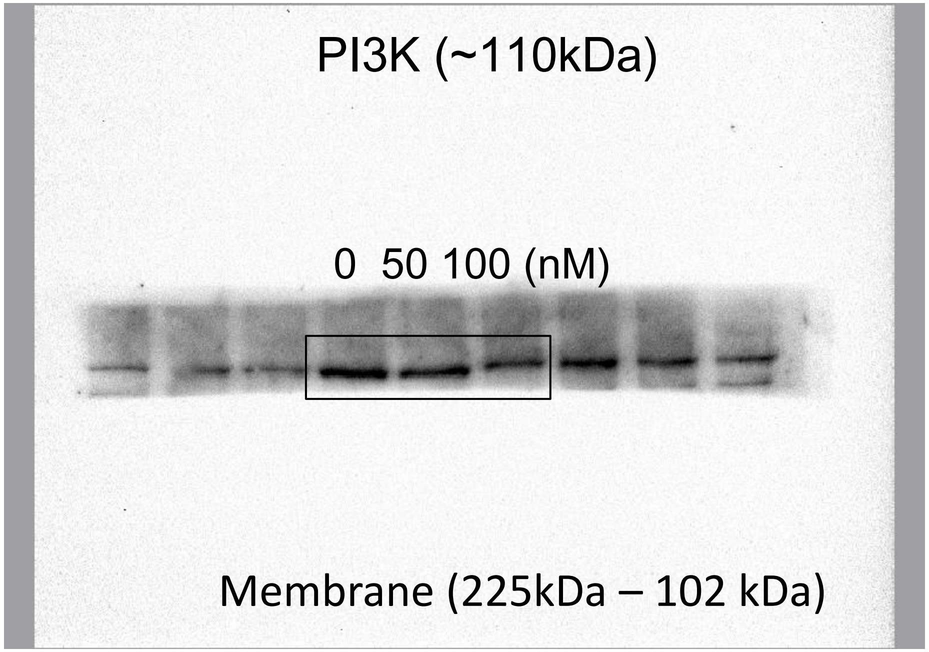

Immunoblots from Figure 5

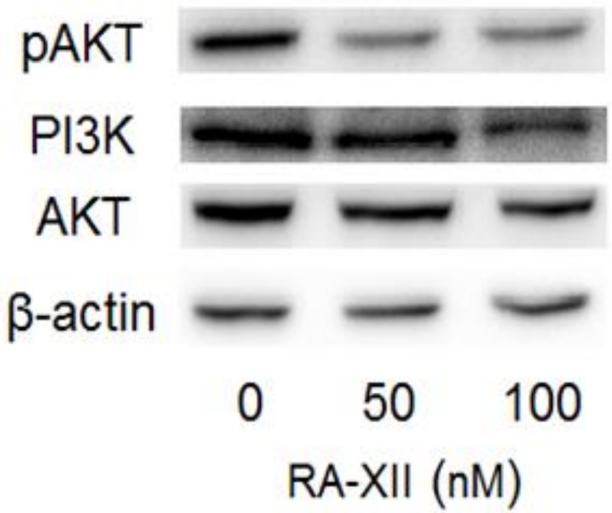

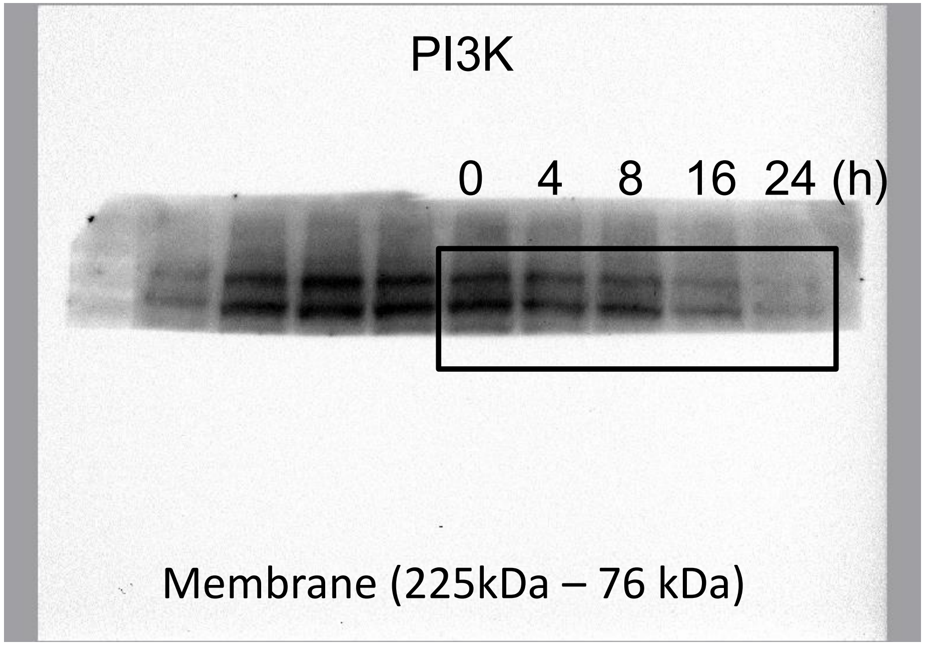

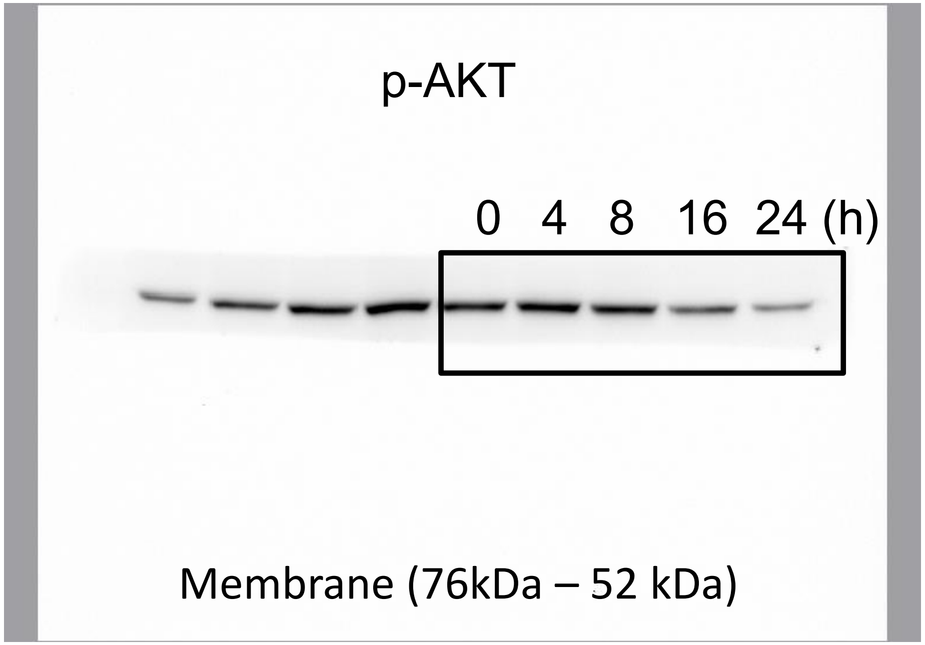

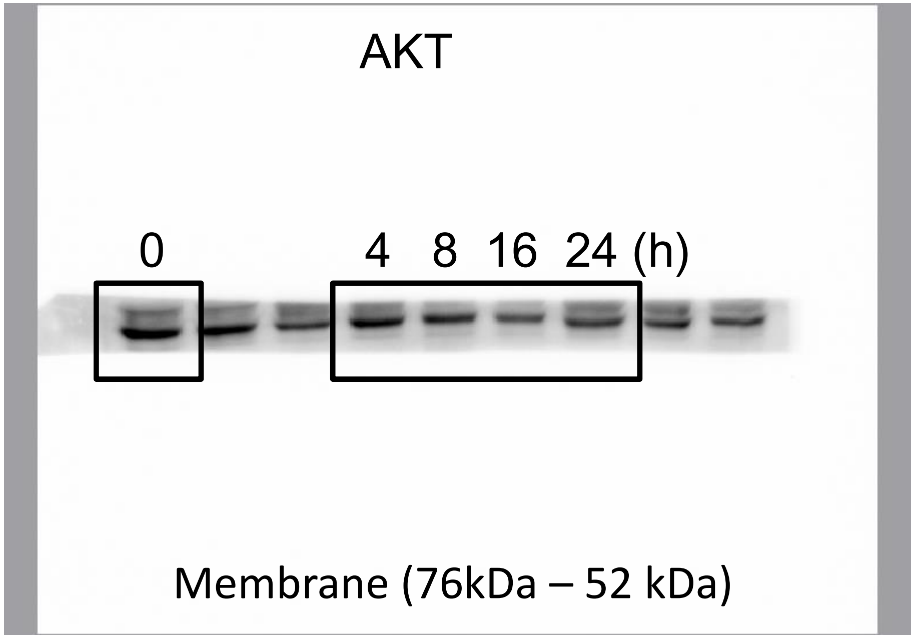

Immunoblots from Figure 5

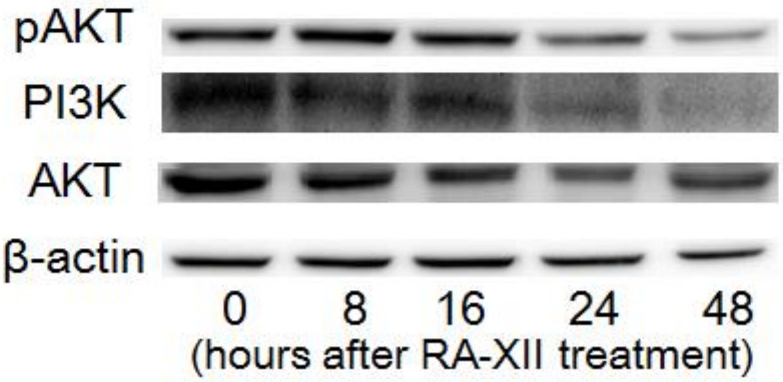

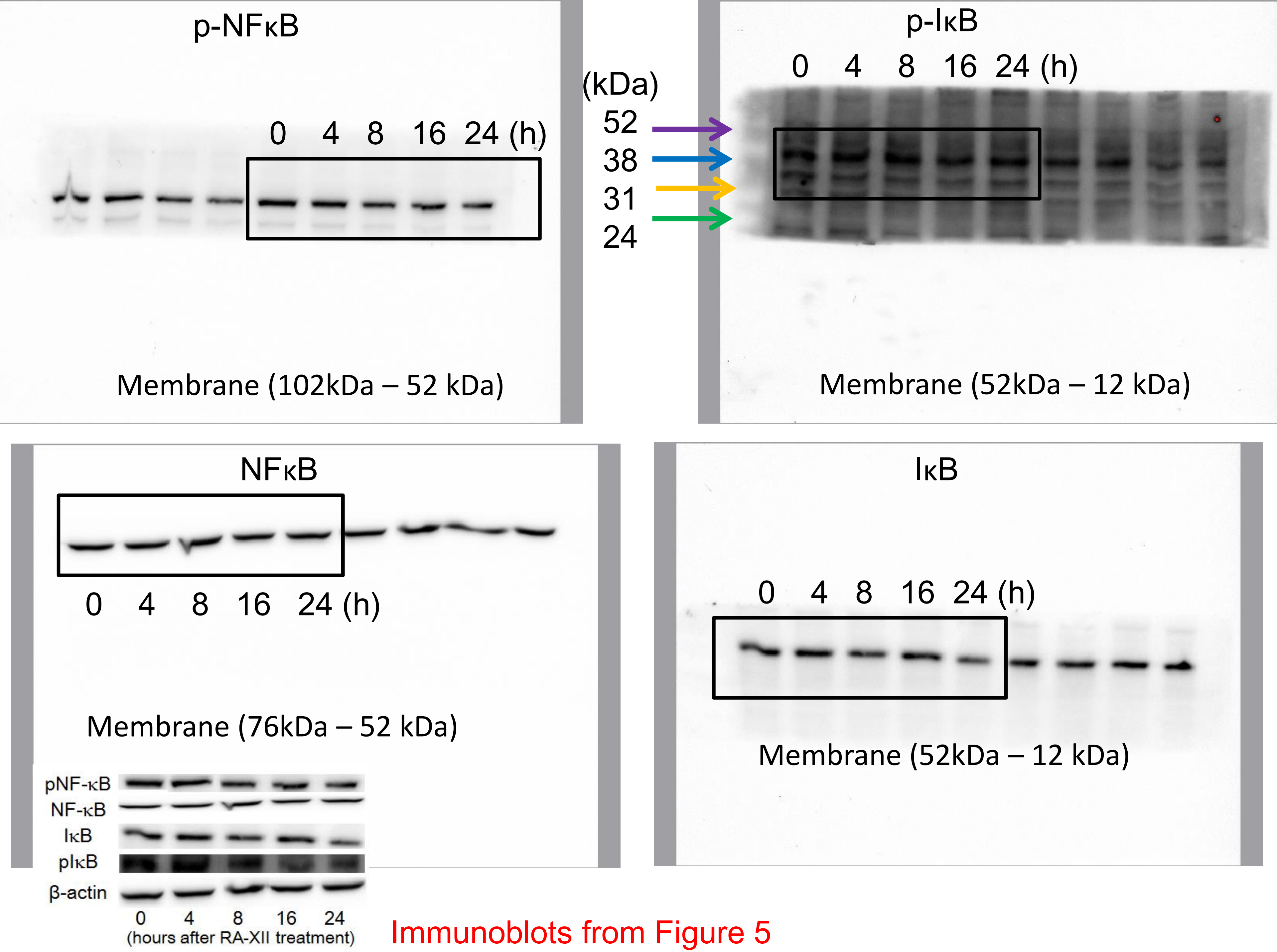

Immunoblots from Figure 5

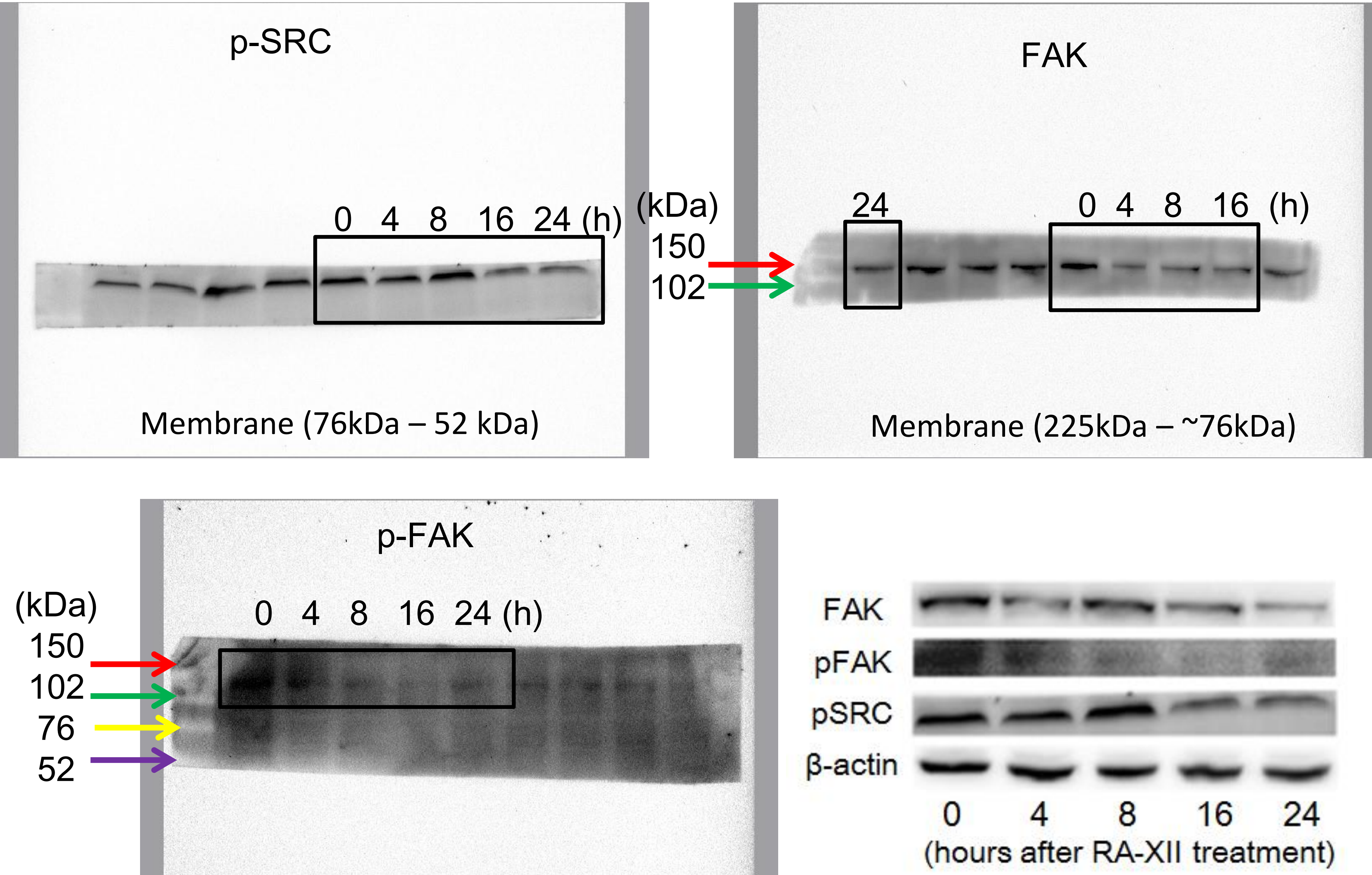

Immunoblots from Figure 5

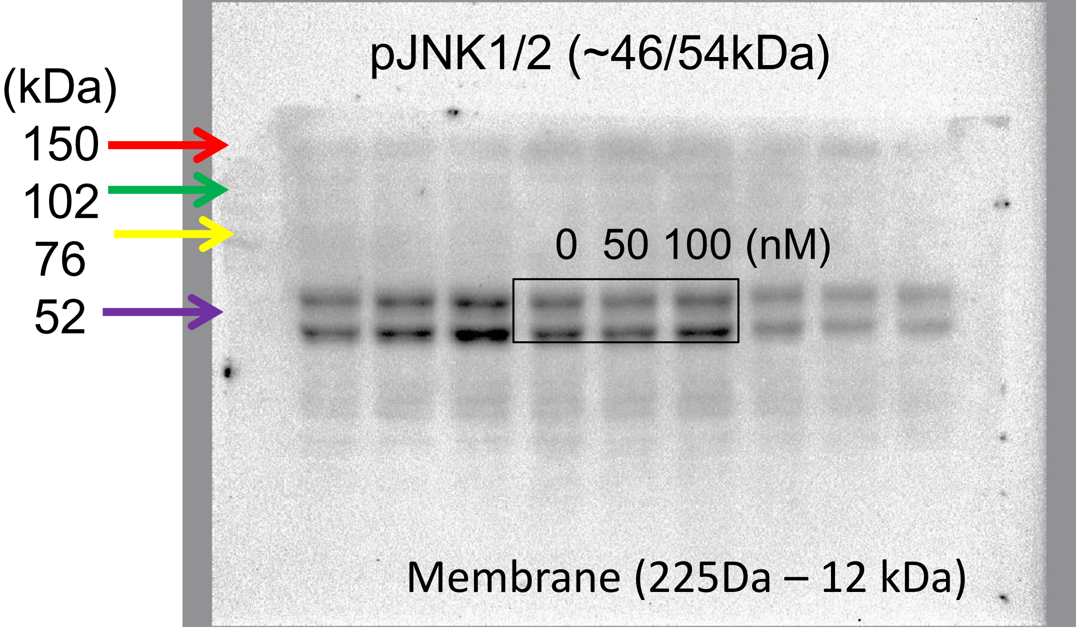

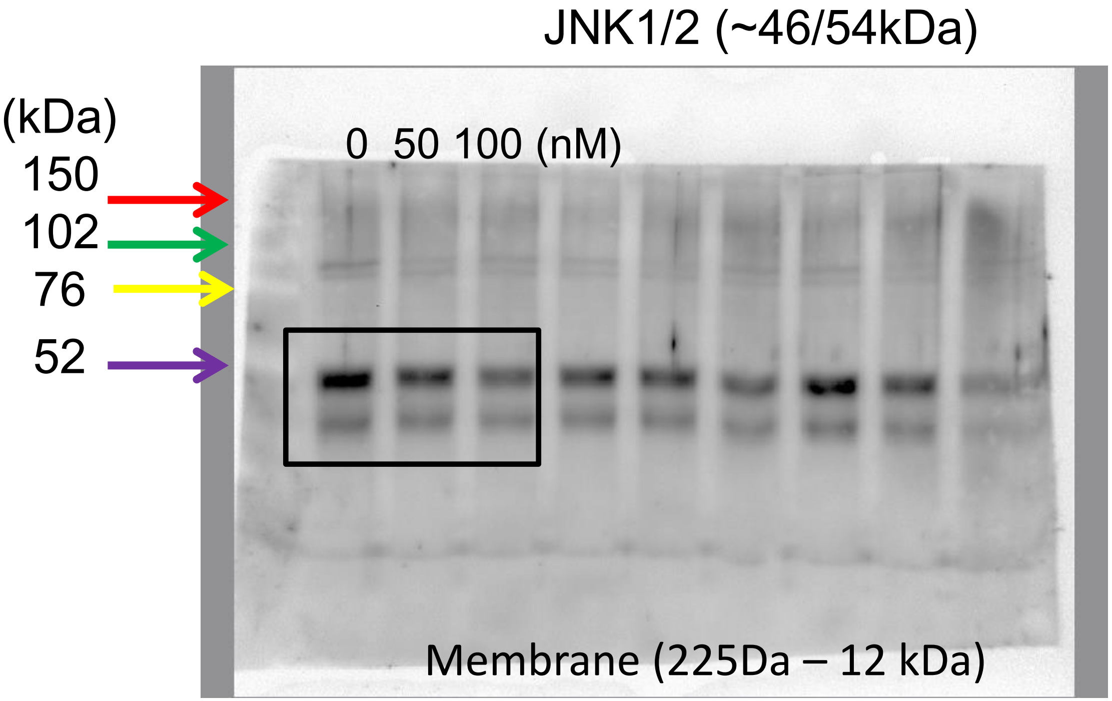

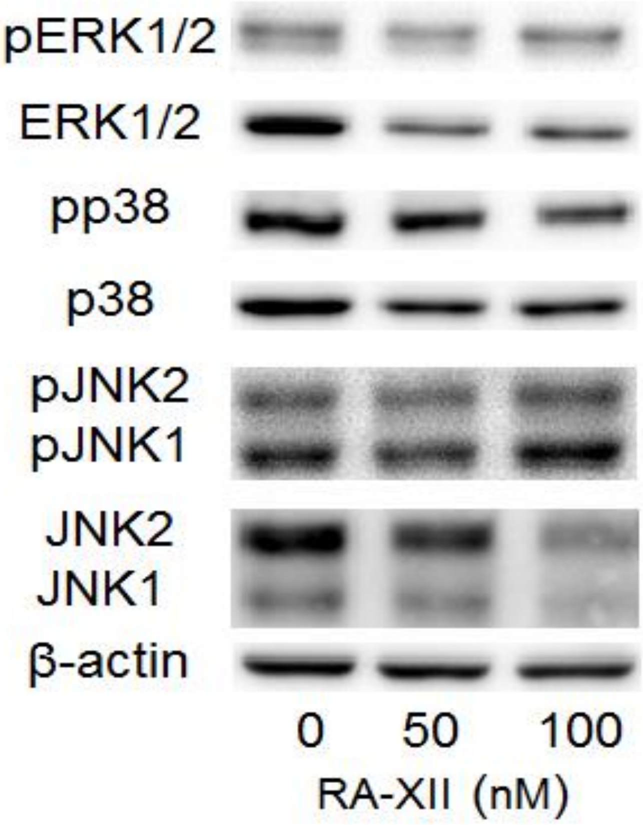

Immunoblots from Figure 5

Immunoblots from Figure 5

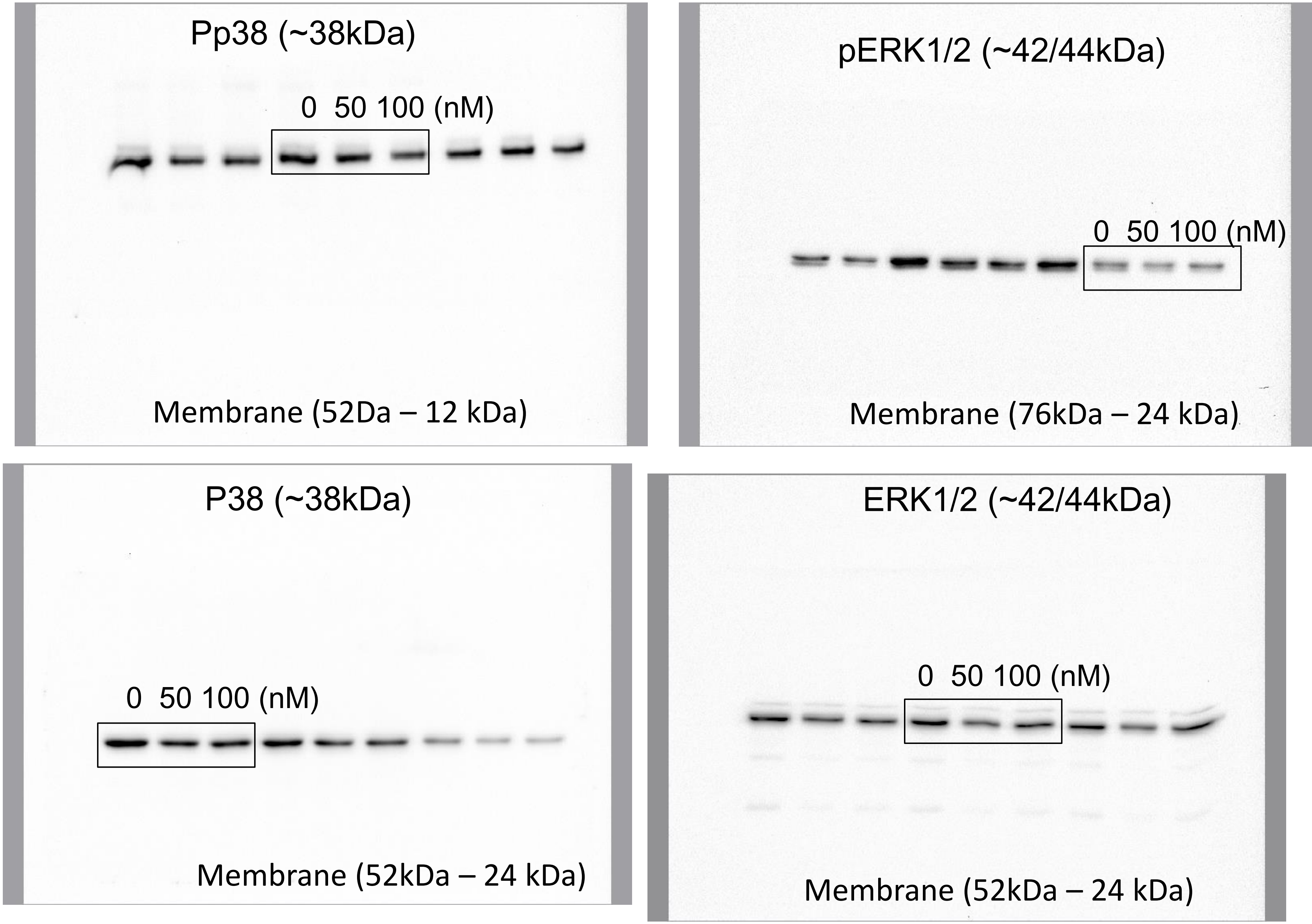

Immunoblots from Figure 5

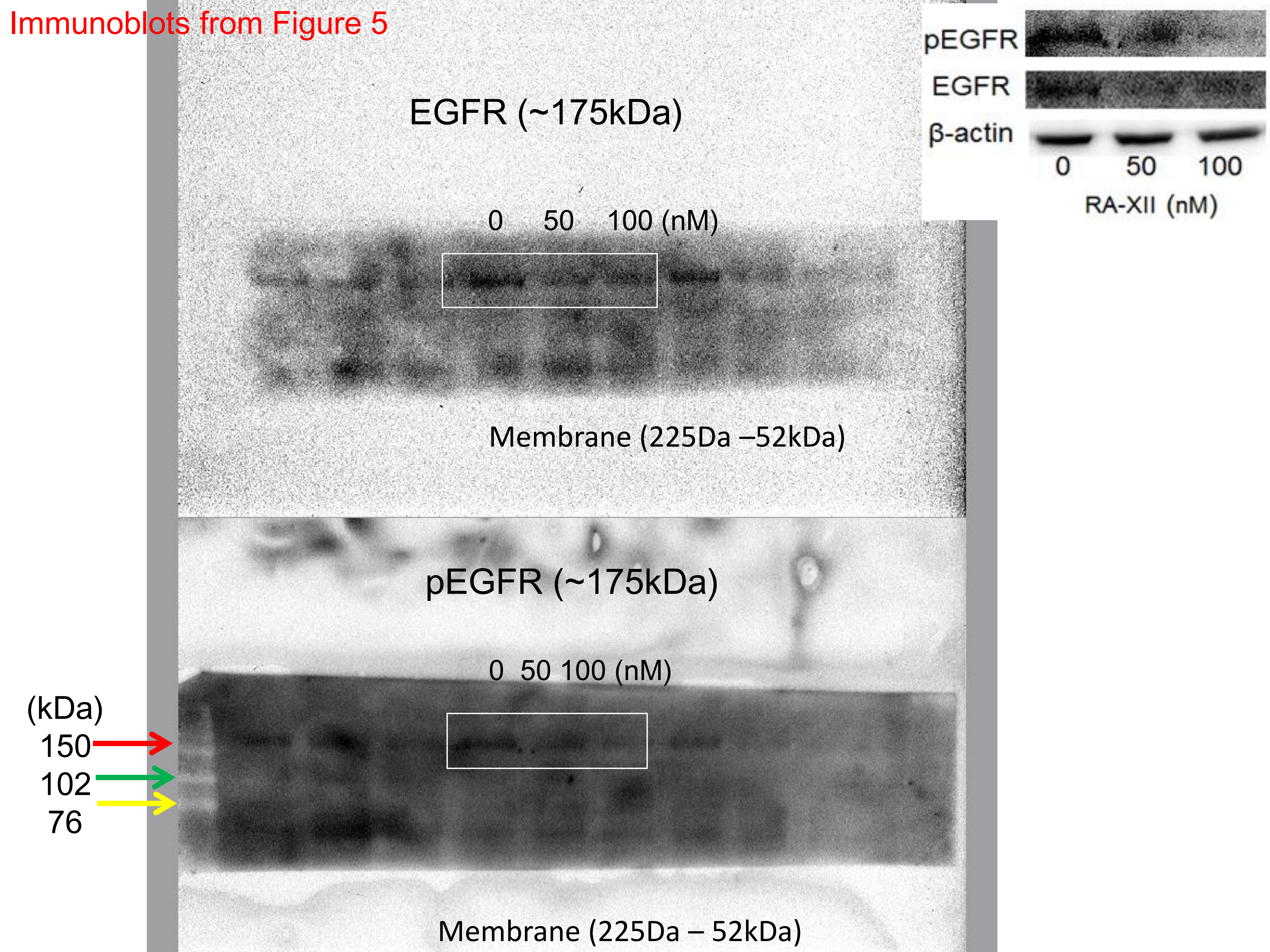

Supplement: Supplementary Information [file srep16985-s1.pdf]
